# An integrated in vitro gastrointestinal digestion and fermentation model as a complementary tool for evaluating feed ingredients in young broilers: a case study with peas

**DOI:** 10.1016/j.psj.2026.108029

**Published:** 2026-10-22

**Authors:** Daphne Michels, Nona De Groof, Sarah H.E. Verkempinck, Evelyne Delezie, Muhammad Zeeshan Akram, Nadia Everaert, Tara Grauwet

**Affiliations:** a KU Leuven, Department of Microbial and Molecular Systems (M2S), Laboratory of Food Technology and Leuven Food Science and Nutrition Research Centre (LFoRCe), Kasteelpark Arenberg 22, PB 2457, 3001 Leuven, Belgium; b KU Leuven, Department of Biosystems (BIOSYST), Nutrition & Animal Microbiota EcoSystems Laboratory, Kasteelpark Arenberg 30, PB 2472, 3001 Leuven, Belgium; c Fisheries and Food, Animal Science Unit, Flanders Research Institute for Agriculture, 9090 Merelbeke-Melle, Belgium

**Keywords:** Protein, in vitro digestion, In vitro fermentation, Broiler

## Abstract

The search for sustainable protein sources in broiler nutrition has increased interest in locally produced ingredients such as peas. However, nutritional evaluation of alternative feed ingredients relies on costly and ethically constrained animal trials, while standardized in vitro models representing the complete gastrointestinal tract of young broilers are lacking. These models should reflect their lower digestive capacity and less complex caecal microbiota. This study developed an innovative in vitro model simulating the complete gastrointestinal tract of young broilers and used peas as a case study to evaluate partial replacement of soybean meal and compare in vitro and in vivo responses. Isoenergetic and isonitrogenous feeds were formulated with 0, 10, 20, or 30% pea inclusion. An in vivo trial with 10-day-old broilers assessed growth performance, protein digestibility, intestinal morphology, caecal fermentation metabolites, and microbiota. In parallel, the same feeds were subjected to the in vitro model, including gastric and small-intestinal digestion and fermentation using a broiler caecal inoculum. Increasing pea inclusion reduced growth performance and protein digestibility and was associated with lower ileal villus height, indicating reduced nutrient utilization at higher raw pea inclusion levels. In vivo caecal short-chain fatty acid and ammonia concentrations were not markedly affected, although ammonia showed a numerical decrease. In vitro, feeds containing 20 or 30% peas showed significantly different digestion kinetics. During in vitro fermentation, pea inclusion increased gas production and acetate and butyrate formation, while reducing ammonia production, suggesting more saccharolytic fermentation. While in vitro results agreed with in vivo protein digestion, caecal fermentation responses were more pronounced in vitro. These findings indicate that raw peas can partially replace soybean meal in young broiler diets and that complementary in vitro and in vivo approaches integrate controlled assessment with physiological confirmation in animals. The model offers controlled assessment before targeted in vivo evaluation, although further research should improve its predictive capacity for caecal fermentation by assessing inoculum representativeness and the selection of soluble and fine-particle digesta fractions for fermentation.

## Introduction

Broiler nutrition relies on the continuous evaluation of, among others, feed ingredients and feed additives to optimize, feed efficiency and cost, nutrient utilization, growth performance, and production sustainability. This is particularly relevant in the search for alternatives to soybean meal (SBM), which remains one of the main protein sources in broiler diets but is associated with environmental concerns, especially in regions where soybean is not produced. Locally available protein sources, such as peas, may contribute to more sustainable feed formulation (Kumar et al., 2025). However, their nutritional value must be carefully assessed, especially in young broilers, where the gastrointestinal tract (GIT) is still developing (Noy and Sklan, 1995).

The nutritional evaluation and the effects of feed ingredients and additives on gut health are mostly assessed using in vivo trials, which provide direct information on animal performance, nutrient digestibility, and gut responses. However, such trials are costly and time-consuming, and they raise ethical concerns related to animal use. In vitro gastrointestinal models offer a complementary approach by enabling rapid, controlled screening of feed ingredients prior to in vivo evaluation (Zaefarian et al., 2021). Such models may support the early selection of promising ingredients or formulations and reduce the number of animal trials required. In addition, in vitro systems can provide mechanistic insights that are difficult to obtain in vivo, including phase-specific digestion kinetics and time-dependent fermentation metabolite evaluations (Michels et al., 2025; Zhao et al., 2026). Additionally, in vivo, it is difficult to quantify the short-chain fatty acids (SCFAs) produced, as they are absorbed by the host.

Despite this potential, standardized in vitro models that adequately represent the gastrointestinal tract of broilers are lacking. Existing approaches often focus on specific digestive compartments or endpoint digestibility measurements, whereas fewer models simulate the complete gastrointestinal tract, including gastric digestion, small-intestinal digestion, and caecal fermentation (Sharma et al., 2022; Zaefarian et al., 2021). Moreover, most available models do not specifically account for the physiology of young broilers. This is an important limitation, as the early post-hatch period is characterized by rapid development of the gastrointestinal tract, increasing digestive enzyme secretion, and development of the microbial composition in the caeca (Noy and Sklan, 1995; Ravindran and Reza Abdollahi, 2021). During the starter phase, digestive enzyme activity and nutrient absorption are still developing, which may reduce the ability to digest complex feed ingredients (Noy and Sklan, 1995). At the same time, the intestinal microbiota is undergoing rapid establishment and succession, making early-life nutrition particularly relevant for later gut function and performance (Ravindran and Reza Abdollahi, 2021). Consequently, feed ingredients that are suitable for older birds may not necessarily be equally suitable for young broilers, and models intended for feed evaluation should reflect this age-specific physiology.

Therefore, the objective of this study was twofold. First, a standardized in vitro model simulating the complete gastrointestinal tract of young broilers was developed and evaluated. The model was designed to mimic gastric digestion, small intestinal digestion, absorption, and caecal fermentation using conditions physiologically adapted to starter broilers. Second, this study aimed to evaluate the potential of raw peas as an alternative protein source to SBM in diets for young broilers using complementary in vivo and in vitro approaches. For this purpose, isoenergetic and isonitrogenous starter feeds were formulated with increasing levels of raw pea inclusion, corresponding to 0, 10, 20, and 30% of the diet, as a partial replacement for SBM. This allowed assessment of raw pea suitability as an alternative protein source in early-life broiler nutrition. At the same time, using the same feeds in vivo and in vitro enabled evaluation and validation of the in vitro system’s ability to reflect the digestive and fermentative responses observed in vivo.

## Materials and methods

### In vivo approach: in vivo performance, protein digestibility, fermentation products, and gut microbiota

#### Ethical statement

The trial was carried out at TRANSfarm, KU Leuven, Bierbeek, Belgium. The feeding trial proposal was approved by the KU Leuven Ethical Committee for Animal Experimentation under project number L006/2024.

#### Bird husbandry and experimental diets

The trial duration was 10 days. A total of 240 1-day-old male Ross 308 chicks were obtained from Belgabroed NV (Merksplas). Chicks were randomly allocated to achieve ten birds per pen and six pens per treatment. Pens were provided with wood shavings, and feed and water were available ad libitum. Isocaloric and isonitrogenous diets were prepared at Instituut voor Landbouw-, Visserij- & Voedingsonderzoek (ILVO, Melle). Four experimental diets were prepared as mash feed: a control diet with soybean meal (SBM) as the main protein source and three diets containing 10%, 20%, or 30% raw-milled peas as an alternative protein source, respectively (Table 1). Birds were fed the respective experimental diets throughout the entire trial period.

**Table 1:** Ingredients and calculated nutritional composition of the control diet and experimental diets containing 10%, 20%, or 30% of peas, respectively (%, DM basis).

Ingrediënt	Control	Pea 10%	Pea 20%	Pea 30%
Wheat	50.85	49.18	40.46	31.74
Soybean meal	30.00	22.00	20.50	19.00
Peas	-	10.00	20.00	30.00
Soybeans	-	7.00	7.00	7.00
Corn	11.51	5.00	5.00	5.00
Animal fat	3.62	2.79	3.07	3.34
Dicalcium phosphate	1.07	1.06	1.07	1.08
Sodium chloride	0.21	0.22	0.23	0.24
Limestone	0.65	0.66	0.65	0.63
Methionine	0.31	0.33	0.35	0.36
L-lysine	0.32	0.30	0.25	0.20
L-threonine	0.14	0.16	0.15	0.14
Sodium bicarbonate	0.15	0.14	0.12	0.11
Phytase	0.10	0.10	0.10	0.10
Coccidiostaticum	0.05	0.05	0.05	0.05
Non-starch polysaccharide enzymes	0.01	0.01	0.01	0.01
Nutrient levels (%)				
Metabolizable energy (MJ/kg)	11.75	11.75	11.75	11.75
Crude protein	21.81	21.68	20.84	21.26
Crude fat	5.65	6.10	6.33	6.56
Crude fiber	2.59	3.16	3.50	3.83
Starch	37.97	36.60	35.26	33.92
Non-starch polysaccharides	12.96	13.66	14.18	14.69
NDF	11.92	10.48	11.19	10.41
ADF	4.78	4.82	5.41	5.55
Digestible Lysine	1.29	1.30	1.31	1.32
Calcium	0.90	0.90	0.90	0.90
Phosphorous	0.56	0.56	0.56	0.56
Sodium	0.13	0.13	0.13	0.13
Chloride	0.24	0.24	0.24	0.24
Potassium	0.91	0.89	0.91	0.92

#### Fiber analysis

Fiber analysis of the diets was performed using the automated fiber analyzer instrument (FiberROC Auto, OPSIS LiquidLINE), with the exception of acid detergent lignin which was done manually. Ashing procedures were performed in a muffle furnace (Select-Horn-TFT, J.P. Selecta) as described in method 5984:2022 by ISO. Crude fiber analysis was based on the Weende crude fiber determination as described in method 978.10 by AOAC (2016). Neutral detergent fiber (NDF), acid detergent fiber (ADF), and acid detergent lignin (ADL) analysis were based on Van Soest et al. (1991). NDF was measured following the AOAC (2005) method 2002.04. ADF and ADL were measured following the AOAC (2016)method 973.18. Samples were homogenized using a knife mill and subsequently passed through two sieves to reach a particle size ranging from 200 µm to 1 mm. Samples with an estimated crude fat content greater than 5% were defatted with acetone prior to analysis.

#### Growth performance and sample collection

Experimental diets were randomized within blocks over the 24 pens in the barn. All birds were weighed on the day of arrival. Birds were weighed after 10 days, as well as their feed intake by measuring the remaining feed in comparison to the initial weight of feed present per diet per pen. These data were used to calculate average daily gain (ADG), average daily feed intake (ADFI), and feed conversion ratio (FCR). On day 10, birds were fed at least 30 min before euthanasia to ensure full intestines for ileal digesta collection. Euthanasia was performed by manual cervical dislocation and exsanguination. Ileal digesta were pooled per pen, and caecal contents were sampled individually of all birds. All ileal and caecal contents were immediately snap-frozen and stored at −80°C until further analysis. Additionally, a segment of the duodenum was sampled from 5 random chicks per pen. Segments were rinsed with phosphate-buffered saline (PBS) and stored in 4% formaldehyde for 48 h, then transferred to 70% ethanol for further preservation.

#### Ileal crude protein digestibility

Protein digestibility was assessed to evaluate the effect of increasing pea inclusion on the amount of protein that can be absorbed from the respective feeds. Ileal digesta were freeze-dried, and silicium and protein content of digesta and the respective diets were determined. Silicium content was determined in triplicate by microwave-assisted digestion followed by ICP-OES analysis. Organic matter in 50 mg of samples was broken down using 65% nitric acid (MILESTONE, Ethos Up, High Performance Microwave Digestion MIL-49010; Analis, Suarlée - Belgium) at 210°C for 20 min. Afterward, samples were diluted to 10 mL with Milli-Q water and filtered (0.45 µm, Chromaphil® A-45/25, Macherey-Nagel, Germany). Si concentrations were determined by ICP-OES analysis (5800 VDV ICP-OES with SPS 4 autosampler and G8496A cooling unit; Agilent Technologies, Diegem, Belgium​​) with radial detection at a wavelength of 336 nm (Staes et al., 2026). Nitrogen content of experimental feeds and digesta was determined using the Dumas method in duplicate (Thermo Scientific FLASH 4000 N analyzer, Italy) and converted to crude protein content using a 6.25 conversion factor. The apparent ileal crude protein digestibility was determined using Equation (1).

(1) $$Digestibility\;\left(\%\right)=\frac{nutrient_{diet}-\left(\frac{SiO_{2diet}}{SiO_{2digesta}}*protein_{digesta}\right)}{protein_{diet}}*100$$

#### Intestinal histomorphology

Intestinal histomorphology was performed as described by Akram et al. (2025). Duodenal segments sampled as described above were embedded in paraffin and sectioned into thin slices using a microtome. Embedded sections were transferred to glass slides and stained with Alcian Blue-Periodic Acid Schiff. Micrographs were taken at a 20x magnification. For each sample, twenty villus-crypt units were analyzed using NDP.view2 software (Hamamatsu Photonics K.K., Hamamatsu, Japan) to obtain villus height (VH), crypt depth (CD), and the VH to CD ratio (VH:CD).

#### Volatile fatty acid analysis

The concentrations of short-chain fatty acids (SCFA; acetate, propionate, butyrate, valerate, and caproate) and branched-chain fatty acids (BCFAs; isobutyrate, isovalerate, and isocaproate) were determined as described by Van Craeyveld et al. (2008) with minor modifications. In short, 250 mg of caecal content was diluted up to a total volume of 350 µL and mixed with 50 µL of 2-methylhexanoic acid (internal standard) and 80 µL of 6 M HCl. Hereafter, samples were placed on ice and regularly mixed to ensure acidification of the volatile FA (VFA). Next, 120 µL 25% NaCl (w/v) and 840 µL tert‑butyl methyl ether were added. Samples were then centrifuged (10.000 g, 5 min, 4°C), after which residual water of an aliquot of the organic layer was removed using anhydrous sodium sulfate. Samples were stored at −20°C until analysis. VFAs were quantified using gas chromatography (Shimadzu Brevis™ GC-2050 System) equipped with an automatic liquid sampler (AOC-30i Series Auto Injector, Shimadzu), a split / spitless injector, a flame ionization detector, and a DB-FFAP capillary column (Agilent J&W GC Columns, 30 m length, 0.25 mm internal diameter, 0.25 μm film thickness). Analyses were carried out using the following oven program: 100°C (3 min) ramped at 4°C·min^−1^ to 165°C, then at 30°C·min^−1^ to 245°C (35 min). The injector and detector temperatures were maintained at 270°C, with a split ratio of 36. Nitrogen was used as carrier gas at linear velocity of 25.0 cm·s^−1^.

#### Ammonia concentration

Ammonia (NH₃) concentrations in the caecal content originating from broilers fed one of the four diets were quantified using a UV-enzymatic ammonia assay kit (AM8870, Biochemical Enterprise) and spectrophotometric detection at 340 nm (Apollo-I, Berthold) in quadruplicate. The assay is based on the glutamate dehydrogenase-catalyzed reaction of NH₃ with 2-oxoglutarate, coupled to the oxidation of NADPH, where the decrease in absorbance is proportional to the NH₃ concentration. Briefly, 200 µL of freshly prepared working reagent was added to a 96-well plate, followed by 20 µL of Milli-Q water, calibrator, or diluted sample. After 3 min of incubation at 37°C, 50 µL of enzyme/NADPH reagent was added to initiate the reaction. Absorbance was recorded at 2 and 7 min after reaction initiation under continuous shaking at 37°C. NH₃ concentrations were calculated from the decrease in absorbance relative to the calibrator and corrected for the sample-specific dilution factor.

#### Microbiota analysis

##### DNA extraction and 16S rRNA gene amplicon sequencing

Approximately 250 mg of caecal content was used to extract DNA with the QIAamp PowerFecal Pro DNA Kit (Qiagen Benelux B.V., Venlo, the Netherlands) according to the manufacturer's instructions. Universal primers 341 F (5′- CCTAYGGGRBGCASCAG-3′) and 806 R (5′-GGACTACNNGGGTATCTAAT-3′) were used to amplify the V3-V4 region of the 16S rRNA gene. Libraries were sequenced on the Illumina NovaSeq 6000 platform by Novogene Co., Ltd (Germany, generating 250 bp paired-end raw reads.

##### Bioinformatics workflow

Raw sequencing data were processed in QIIME2 (v2025.04), where reads underwent quality control, trimming, and demultiplexing using the default pipeline settings. Amplicon sequence variants (ASVs) were inferred following the removal of low-quality reads with the DADA2 algorithm. Taxonomic classification of ASVs was performed using a Naïve Bayes classifier trained on the SILVA reference database (release 138.2).

Subsequent statistical analyses were conducted in R (v4.2.3; R Foundation for Statistical Computing, Vienna, Austria) after importing the QIIME2 output. Alpha diversity was estimated using the Chao1, Shannon, and Simpson indices with the phyloseq package (v1.40.0) following rarefaction to the minimum sequencing depth. Differences in alpha diversity among groups were evaluated using the Kruskal–Wallis test. Community dissimilarity (beta diversity) was assessed based on Bray–Curtis distances and visualized by principal coordinates analysis (PCoA). Statistical differences in microbial community composition were tested by permutational multivariate analysis of variance (PERMANOVA) with 9,999 permutations using the vegan package (v2.7.6). Prior to PERMANOVA, homogeneity of multivariate dispersion was evaluated using the vegan package (v2.7.6). Following significant PERMANOVA results, a pairwise PERMANOVA comparison was performed using the pairwiseAdonis package (v0.4.1;GitHub) and p-values were adjusted using the Benjamini-Hochberg FDR correction (Benjamini and Hochberg, 1995).

Differentially abundant genera were identified using linear discriminant analysis effect size (LEfSe) implemented in the microbiome package (v1.18.0). Taxa with an LDA score ≥ 2.0 and P < 0.05 were considered significantly enriched. To account for multiple comparisons, P-values were adjusted using the Benjamini–Hochberg false discovery rate (FDR) correction (Benjamini and Hochberg, 1995), and significant taxa were presented as log10-transformed LDA scores.

##### Statistical analysis of in vivo performance and digestibility data

Both performance and protein digestibility data were analyzed using ANOVA (JMP 19). Factors taken into account in analyzing the effect of dietary treatment on the performance of the birds were body weight gain (BWG), feed intake (FI), and FCR during the entire study period.

### In vitro approach: In vitro simulation of the young broiler GIT of experimental diets

#### Materials

Samples of the experimental diets described above were stored at −20°C until further analysis. Analytical and HPLC-grade reagents were acquired from Sigma-Aldrich (Diegem, Belgium), besides NaCl, H_2_SO_4_, NaHCO_3_, KH_2_PO_4_, trimethylamine, and ethanol (Fisher Scientific, Merelbeke, Belgium); HCl, diethyl ether, and iso-propanol (VWR, Leuven, Belgium); KCl, MgCl_2_(H_2_O)_6,_ and heptane (Acros Organics, Geel, Belgium), Total Starch Kit (Megazyme, Bray, Ireland), sodium potassium tartrate (Acros Organics, Geel, Belgium).

#### Static in vitro simulation of the gastrointestinal tract of young broilers

Static in vitro digestion was performed on the experimental diets. This study specifically focused on the potential of peas as an alternative protein source in young broilers. In this context, literature has already clearly described the physiological differences (e.g., enzymatic secretions) between starter, grower, and finisher broilers (Noy and Sklan, 1995; Ravindran, 2013; Sklan and Noy, 2000; Svihus, 2014). Therefore, it was aimed to develop and propose a standardized in vitro model simulating the GIT of young broilers from proventriculus to caeca. Moreover, most studies only focus on isolated parts of the GIT. Therefore, it was aimed to gain large-scale insights by studying the GIT in vitro from proventriculus/gizzard to caeca. The protocol was based on Michels et al. (2025) and Zhao et al. (2026) with slight adaptations in enzymatic activities, GIT transit times, temperature, and caecal starting material as described by in vivo literature to specifically account for the young broiler physiology. More specifically, protease activities of pepsin, trypsin, and chymotrypsin were based on both in vivo and in vitro literature described by Bryan et al. (2018b), Kadhim et al. (2014), Noy & Sklan (1995), and O’Sullivan et al. (1992). In vivo data (Kadhim et al., 2014; Noy and Sklan, 1995; O’Sullivan et al., 1992) were converted to be expressed in U/g or U/mL of chyme and to estimate final extents of digestibility in young broilers. Amylase activity was determined based on data reported by Noy & Sklan (1995), Sklan & Noy (2000), and Wiseman et al. (2000), indicating that starch is digested to relatively high extents even in young broilers. Bile salt concentrations were determined based on data reported by Green & Kellogg (1987). Additionally, currently existing in vitro protocols describing broiler digestion (Bryan et al., 2018; Ebsim, 2013; Martinez-Haro et al., 2009; Menezes-Blackburn et al., 2015; Weurding et al., 2001; Zyła et al., 2000) were assessed to evaluate their reasoning and selection of in vitro parameters. Lastly, GIT transit times, pH values, and body temperature were selected based on the previously described in vitro protocols and data reported by Lee et al.(2017), Ravindran (2013), Svihus (2011). An overview of the conditions used to mimic the young broiler GIT in this research is depicted in Fig. 1.

**Fig. 1:**
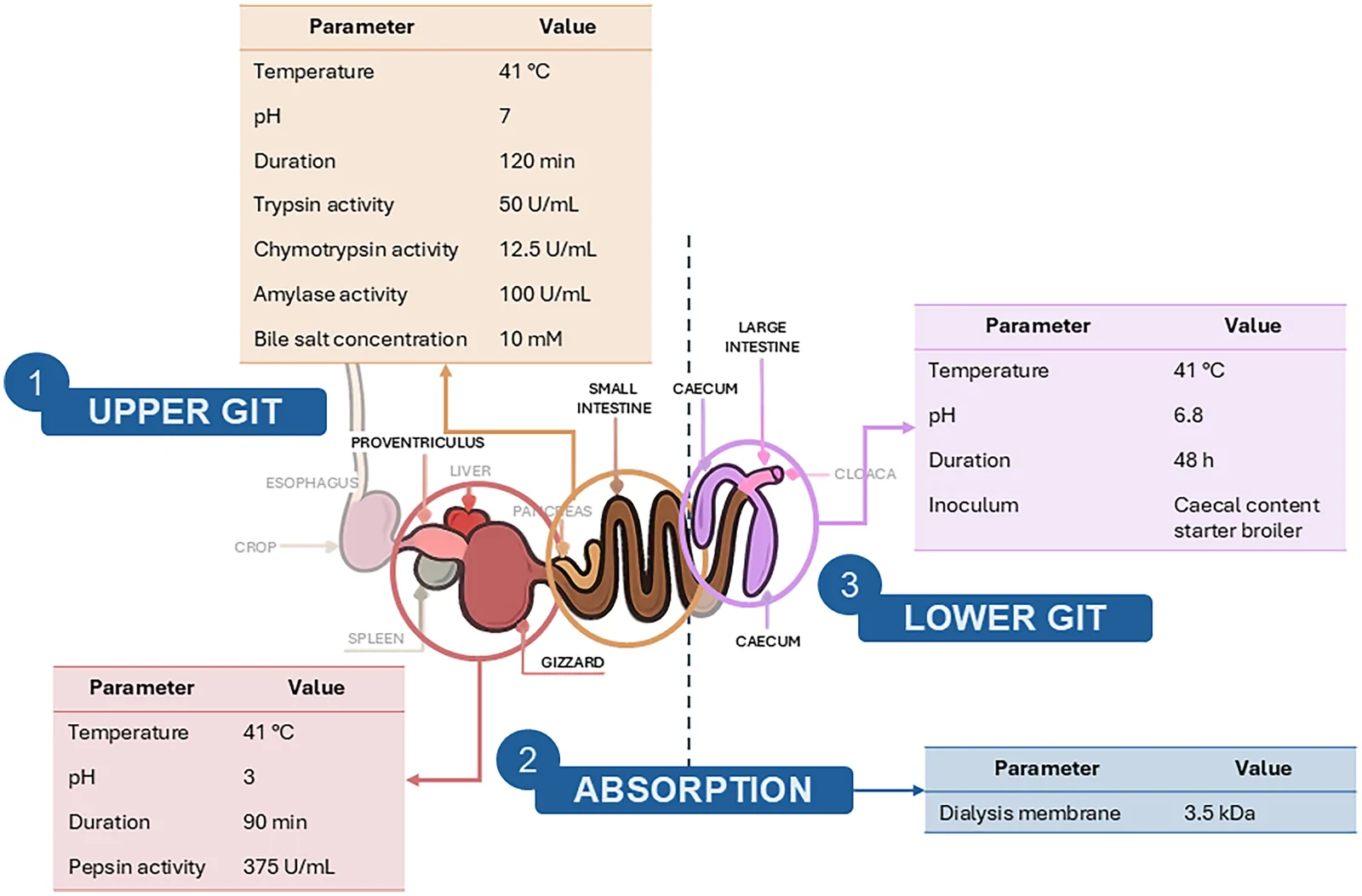
Overview of the conditions used to simulate the young broiler gastrointestinal tract (GIT) in the upper GIT, absorption, and the lower GIT. Conditions were adapted according to *in vivo* literature (Bryan et al., 2018; Ebsim, 2013; Kadhim et al., 2014; Martinez-Haro et al., 2009; Menezes-Blackburn et al., 2015; Noy and Sklan, 1995; O’Sullivan et al., 1992; Sklan and Noy, 2000; Weurding et al., 2001; Wiseman et al., 2000; Zyła et al., 2000).

##### In vitro simulation of the upper GIT

First, the gastric phase (GP) was mimicked by combining 0.625 g of ground feed (< 1 mm) with 1.25 mL Milli-Q water, 2 mL simulated gastric fluid, 50 µL CaCl₂ (7.5 mM), and 125 µL pepsin (375 U/mL chyme). The pH was adjusted to 3 using 6 M HCl, after which Milli-Q water was added to reach a final digestion volume of 5 mL. Samples were incubated at 41°C under end-over-end rotation for 90 min. To obtain the sample corresponding to the start of the gastric phase (GP 0), the mixture was subjected to a heat shock at 98°C prior to pepsin addition to ensure immediate enzyme inactivation. After 90 min, a second sample representing the end of the gastric phase (GP 90) was heat-shocked at 98°C to inactivate pepsin and terminate digestion.

Subsequently, the small intestinal phase (SIP) was simulated by supplementing the gastric digesta with 2 mL simulated intestinal fluid, 400 µL CaCl₂ (7.5 mM), 750 µL bile salt solution (10 mM in chyme), and 1.25 mL pancreatic enzyme solution containing trypsin and chymotrypsin. Enzyme activities were adjusted to reflect young broiler conditions (100 U/mL amylase, 50 U/mL trypsin, and 12.5 U/mL chymotrypsin). The pH was adjusted to 7 using 1 M NaOH, then Milli-Q water was added to reach a final volume of 10 mL. Incubation was carried out at 41°C with continuous end-over-end rotation. Digestion was terminated at predefined time points (0, 5, 10, 15, 20, 30, 45, 60, 90, and 120 min) by heat treatment (98°C, 5 min) to inactivate enzymatic activity. In both the GP and SIP, a digest blank was prepared that underwent the same steps as described above, but lacked feed sample.

Following digestion, samples were centrifuged (2000 × g, 5 min, 25°C) to separate supernatant and pellet fractions. The supernatant was rapidly frozen in liquid nitrogen and stored at −40°C until further analysis.

##### Quantitative analysis of protein digestion products in the upper GIT

Readily bio-accessible protein in the digest supernatant was quantified using the spectrophotometric o-phthaldialdehyde (OPA) method as described by (Pälchen et al., 2021). This method quantifies α-amino groups of amino acids (AA) and peptides released upon hydrolysis in the in vitro upper GIT.

The total amount of amino groups present in the undigested sample (NH_2total_) was determined by hydrolyzing 5 mg of feed in 1 mL of 6 N HCl (24 h, 110°C) in triplicate. After hydrolysis, the acid was neutralized using 1 mL of 6 N NaOH and further diluted to 10 mL with Milli-Q water. Hereafter, samples were filtered through 0.25 µm syringe filters. Digests and digest blanks (i.e., NH_2initial_) were first treated with 3.2% trichloroacetic acid (TCA) to precipitate larger non-bioaccessible peptides. TCA-precipitated samples were centrifuged (10,000 g, 25°C, 30 min; Microfuge 22R, Beckman Coulter, US) and supernatants were hydrolyzed in 9 N HCl (16 h, 110°C). Similarly, acid was neutralized using 1 mL of 9 N NaOH and further diluted to 10 mL with Milli-Q water and filtered (0.25 µm), resulting in the NH_2TCA,hydro_ fraction.

OPA reagent was prepared fresh on the day of analysis as described by Zahir, Fogliano, and Capuano (2018). Digestion products were converted to serine equivalents using a serine standard curve (12.5 – 100 mg/mL). Each sample was analyzed in duplicate by measuring absorbance at 340 nm after a 2 min dark incubation with the OPA reagent. The extent of protein digestion at time *t* was calculated using equation (2).

(2) $$Hydrolysed\;readily\;bioaccessible\;protein\;\left(\%\right)=\frac{NH_{2TCA,hydro}\left(t\right)-NH_{2initial}}{NH_{2total}}*100$$

##### Statistical analysis of kinetic digestion data

Protein hydrolysis kinetics were integrated using nonlinear regression analysis to model *in vitro* hydrolysis kinetics. Different models were considered to fit the time-dependent response of protein hydrolysis as assessed by their R^2^ and R^2^_adj_. After model discrimination, a fractional conversion model was applied (Duijsens et al., 2022). The equations representing these models are displayed in equation (3).

(3) $$C\left(t\right)=\;C_{f}+\left(C_{0}-C_{f}\right)*e^{-k*t}$$

The final extent is represented by *C_f_* (%), the plateau value of protein hydrolysis at an infinite time. The rate constant of macronutrient hydrolysis is *k* (min^−1^). Kinetic parameters *C_f_* and *k* were estimated simultaneously using a nonlinear regression procedure (SAS version 9.4, SAS Institute, Inc., Cary, NC, USA). Differences between digestion kinetics of different diets estimated with the fractional conversion model were determined based on their joint confidence regions (JCR) for both jointly estimated kinetic parameters (α = 0.01) (Pallares Pallares et al., 2018).

##### In vitro absorption simulation

Substrate for all diets to simulate in vitro batch fermentation was prepared as described in the section above, simulating SIP 120 at 80 mL to obtain sufficient substrate. Bioaccessible nutrients, bile salts, and enzymes were removed from the digest using a 3.5 kDa dialysis membrane (Fako et al., 2026), submerged in continuously stirring Milli-Q water at 4°C. Water was refreshed at 2 h intervals up to 8 h, and at 4 h intervals between 8 h and 72 h. The final undigested fraction remaining in the dialysis membrane was subsequently freeze-dried for 24 h and stored in a desiccator until further use. This dialysis step served as an operational separation to remove low-molecular-weight, potentially absorbable digestion products and bile components from the digest, generating an undigested fraction representative of the material available for subsequent caecal fermentation. Within the present experimental setup, this provides a controlled and reproducible approach to distinguish material removed during small-intestinal digestion from the fraction retained for hindgut fermentation, while recognizing that selective intestinal transport and physiological transit are not reproduced.

##### In vitro simulation of the lower GIT

In vitro batch fermentation was performed on the undigested fraction obtained after simulated digestion in the upper GIT and absorption. For this purpose, caecal inoculum of 2-week-old Ross 308 broilers was used. This inoculum originated from pooled caecal content with two biological replicates of 1:1 male and female broilers from a previous trial, fed a wheat- and SBM-based diet. The use of caecal content of 14-day-old broilers is justified as literature indicates that the most pronounced microbial succession occurs during the first week post-hatch, whereas the microbiota progressively matures thereafter (Kers et al., 2022; Yin et al., 2023), although inoculum originating from 10-day-old broilers would have been desired. The inoculum was suspended in anaerobically prepared phosphate-buffered saline solution (pH 7.2) at a ratio of 1:9 (w/v) to achieve a 10% inoculum concentration and homogenized with a stomacher (Mixwel+, Alliance Bio Expertise). Hereafter, suspensions were filtered through a 200 µm sieve (Retsch, Germany) to remove particles. The fermentation medium (pH 6.8) consisted of 1.35 g/L Na_2_HPO_4_, 1.47 g/L KH_2_PO_4_, 0.14 g/L MgSO_4_⋅7H_2_O as macro minerals, 12 mg/L MnCl_2_⋅4H_2_O, 15.84 mg/L CaCl_2_⋅2H_2_O, 1.20 mg/L CoCl_2_⋅6H_2_O, 0.96 mg/L FeCl_3_⋅6H_2_O as essential minerals for microbial growth, 8.3 g/L NaHCO_3_, 0.98 g/L NH_4_Cl for pH stabilization and nitrogen supply, 0.3 g/L Na_2_S⋅9H_2_O as a reducing agent, and 1 mg/L resazurin as an oxygen indicator. For each diet and fermentation time point, independently prepared and incubated fermentation replicates constituted the experimental units. For each replicate, 100 mg of the undigested fractions from each diet was transferred to a 50 mL serum vessel in triplicate for each fermentation time point (6, 12, and 24 with the exception of 48 h, for which four replicates were prepared, resulting in n = 3 and n = 4, respectively, for statistical analysis) and mixed with 2 mL inoculum and 8 mL buffer inside an anaerobic chamber (Whitley A35 Workstation, Don Whitley Scientific). Additionally, for each time point, 3 blank fermentation replicates were prepared (without substrate). Fermentation vessels were sealed with a septum and crimped aluminum cap, and incubated in a water bath at 41°C with a stirring shaft speed of 80 rpm for 48 hours under anaerobic conditions. After 6, 12, 24, and 48 hours of fermentation, three fermentation replicates (four for 48 h) per diet were placed in an ice bath to halt the fermentation process, and four 2 mL aliquots were taken from each vial, snap-frozen, and stored at −80°C until further analysis.

### *Analysis of gas production kinetics and fermentation products in the lower GIT*

#### Gas production kinetics

Gas pressure was registered at 2, 5, 8, 12, 16, 20, 24, 36, and 48 hours (n = 4 technical replicates) throughout fermentation using a digital pressure gauge (DG25, Ashcroft) equipped with a 20 G luer-lock needle (Henke Sass Wolf). The registered gas pressure was converted to a cumulative gas volume (mL/g) and fitted with the monophasic Groot model, as described in equation (4) The model was fitted using nonlinear least-squares regression, after which cumulative gas volume was plotted against fermentation time. Parameters *A_i_* and *B_i_* denote the asymptotic gas production (mL/g protein source) and the time (h) at which half of this asymptotic value is reached, respectively. *C_i_* is a dimensionless constant determining the sharpness of the transition in the profile. The maximum gas production rate (*R_max_,* mL/h), and the time at which *R_max_* occurred (*t_Rmax_,* h) were calculated.

(4) $$GP_{c}=\frac{A_{1}}{1+{\left(\frac{B_{1}}{t}\right)}^{C_{1}}}+\frac{A_{2}}{1+{\left(\frac{B_{2}}{t}\right)}^{C_{2}}}\;t\leq t_{GP_{s}}$$

#### Metabolite formation during in vitro batch fermentation

SCFA and ammonia production in in vitro fermentation samples at 6, 12, 24, and 48 h were determined as described in the method section of the in vivo trial above. Microbiota were analyzed as described before, with small adaptations. 350 µL fermentation juice was used for SCFA extraction instead. Sample dilutions for ammonia measurement were directly prepared with the fermentation juice using saline solution.

## Results

### In vivo broiler trial

#### Effect of pea on apparent ileal crude protein digestibility and growth performance

Growth performance parameters measured during the 10-day trial period, such as BW, BWG, and FCR, are summarized in Table 2. Birds were divided over different treatments and pens, ensuring that their starting weight was not significantly different (P > 0.01). Over the 10-day trial period, the feed intake was not significantly different between birds fed different diets (P > 0.05). However, a clear significant decrease in final BWG after 10 days was registered in broilers fed experimental diets with increasing quantities of pea incorporated in comparison to broilers fed the soybean meal control diet. More specifically, birds fed the Pea 30% diet showed a significantly lower BW and gained significantly less weight in comparison to broilers fed the control diet (P < 0.05). However, these differences did not result in significantly different FCRs. Additionally, AID also showed a substantial downward trend from the control diet to diets containing increasing amounts of pea, although these differences were not significant (P > 0.05).

**Table 2:** Growth performance and apparent ileal protein digestibility (AID) of young broilers (n = 6 pens) from d 0 to 10 fed diets with increasing concentrations of pea (10%, 20%, and 30%, respectively) compared to a soybean meal control diet.

	Control	Pea 10%	Pea 20%	Pea 30%
**BW at day 1 (g)**	41.1 ± 0.3^a^	41.3 ± 0.3^a^	41.2 ± 0.1^a^	41.0 ± 0.3^a^
**BW at day 10 (g)**	233.0 ± 11.2^a^	224.3 ± 7.0^a^^,^^b^	220.0 ± 5.2^a^^,b^	215.7 ± 8.3^b^
**BWG (g/10d)**	187.2 ± 8.3^a^	175.4 ± 7.2^a^^,^^b^	174.8 ± 7.8^a^^,b^	166.9 ± 5.8^b^
**FI (g/bird/10d)**	248.0 ± 5.6^a^	250.2 ± 28.6^a^	239.7 ± 6.1^a^	237.2 ± 9.5^a^
**FCR**	1.3 ± 0.0^a^	1.4 ± 0.2^a^	1.4 ± 0.0^a^	1.4 ± 0.1^a^
**AID of CP**	61.03 ± 2.92^a^	60.77 ± 3.53^a^	57.07 ± 4.86^a^	55.98 ± 6.49^a^

a–b Means within the same row with no common superscript differ significantly (p < 0.05).

#### Pea inclusion altered ileal histomorphology

Ileal histomorphological parameters (VH, VH:CD, and the number of goblet cells) are depicted in Table 3. Overall, there were few significant differences in ileal histomorphological characteristics between different treatment groups. However, a significant downward trend in villus height was observed from the control group toward the pea 30% group.

**Table 3:** Ileal histological characteristics of broilers fed diets with increasing concentrations of pea (10%, 20%, and 30%, respectively) compared to a soybean meal control diet.

Item^1^	Control	Pea 10%	Pea 20%	Pea 30%
**VH (µm)**	502.8 ± 10.7a–b	523.1 ± 11.2^a^	502.7 ± 15.4a–b	472.7 ± 6.7a–b
**VH:CD**	2.7 ± 0.1a–b	2.7 ± 0.1a–b	2.8 ± 0.1a–b	2.8 ± 0.1a–b
**Goblet cells (#)**	103.0 ± 4.5a–b	108.4 ± 6.5a–b	109.3 ± 6.4a–b	89.0 ± 5.5a–b

a–b Means within the same row with no common superscript differ significantly (p < 0.05).

1 VH = villus height, CD = crypt depth, VH:CD = ratio of VH to CD.

#### Effect of pea on caecal SCFA, BCFA, and ammonia production

Fig. 2 depicts the concentrations of acetic, propionic, butyric, iso-butyric, iso-valeric, and valeric acid measured in caecal contents of broilers at the end of the trial period. The acetic acid concentration was highest across all treatments, followed by butyric acid and propionic acid. Iso-butyric, iso-valeric, and valeric acid concentrations were all lower than 0.2 mg/g caecum content. No significant differences were observed in SCFA and BCFA production across treatments (P > 0.05). Similarly, ammonia concentrations (Fig. 3) across treatments were not significantly different (P > 0.05), although a substantial numerical drop in ammonia concentration from approximately 0.35-0.37 to 0.27 g/ g caecum content was seen in the Pea 30% treatment in comparison to other treatments.

**Fig. 2:**
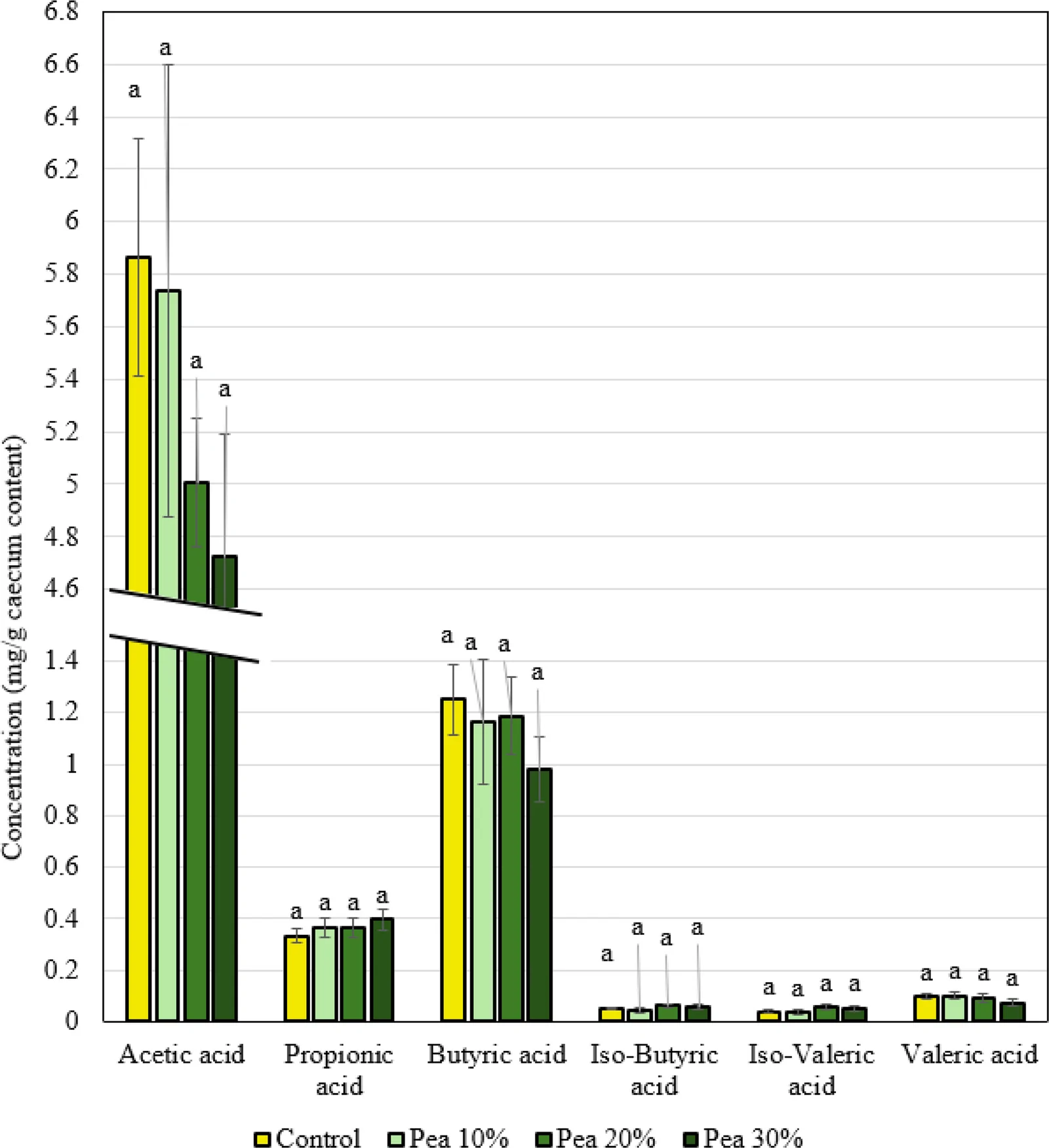
Caecal SCFA and BCFA concentration across the four experimental diets (i.e., control, Pea 10%, Pea 20%, Pea 30%). Significant differences (P < 0.05) between experimental diets are indicated with different letters.

**Fig. 3:**
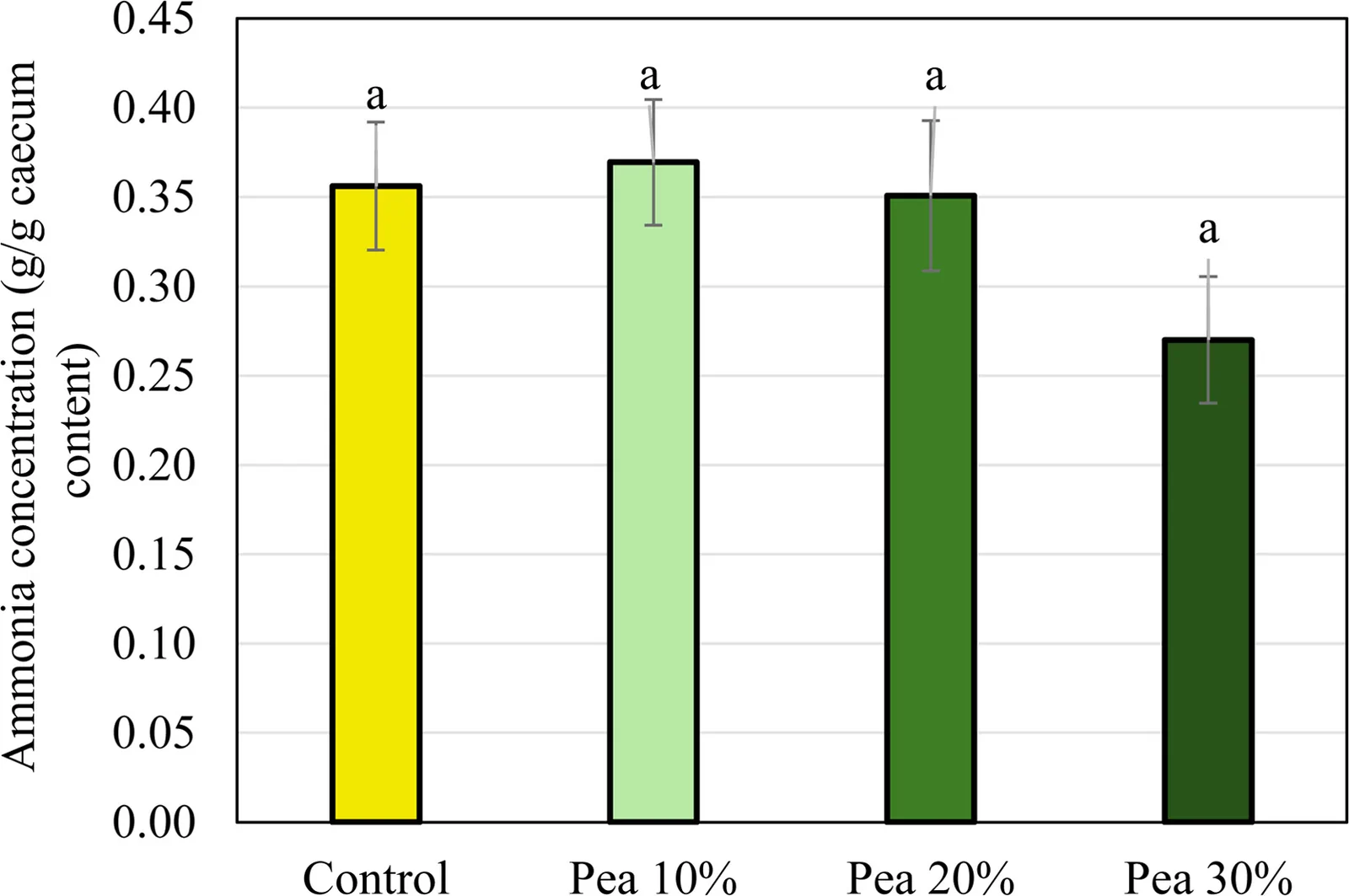
Caecal ammonia concentration across the four experimental diets (i.e., control, Pea 10%, Pea 20%, Pea 30%). Significant differences (P < 0.05) between experimental diets are indicated with different letters.

#### Higher pea inclusions altered microbial composition of caecal content

***Alpha diversity.*** Three parameters were determined to evaluate the α-diversity of the four experimental treatments: the Chao1, Simpson, and Shannon indices (Fig. 4). None of the three indices differed significantly across the four experimental diets.

**Fig. 4:**
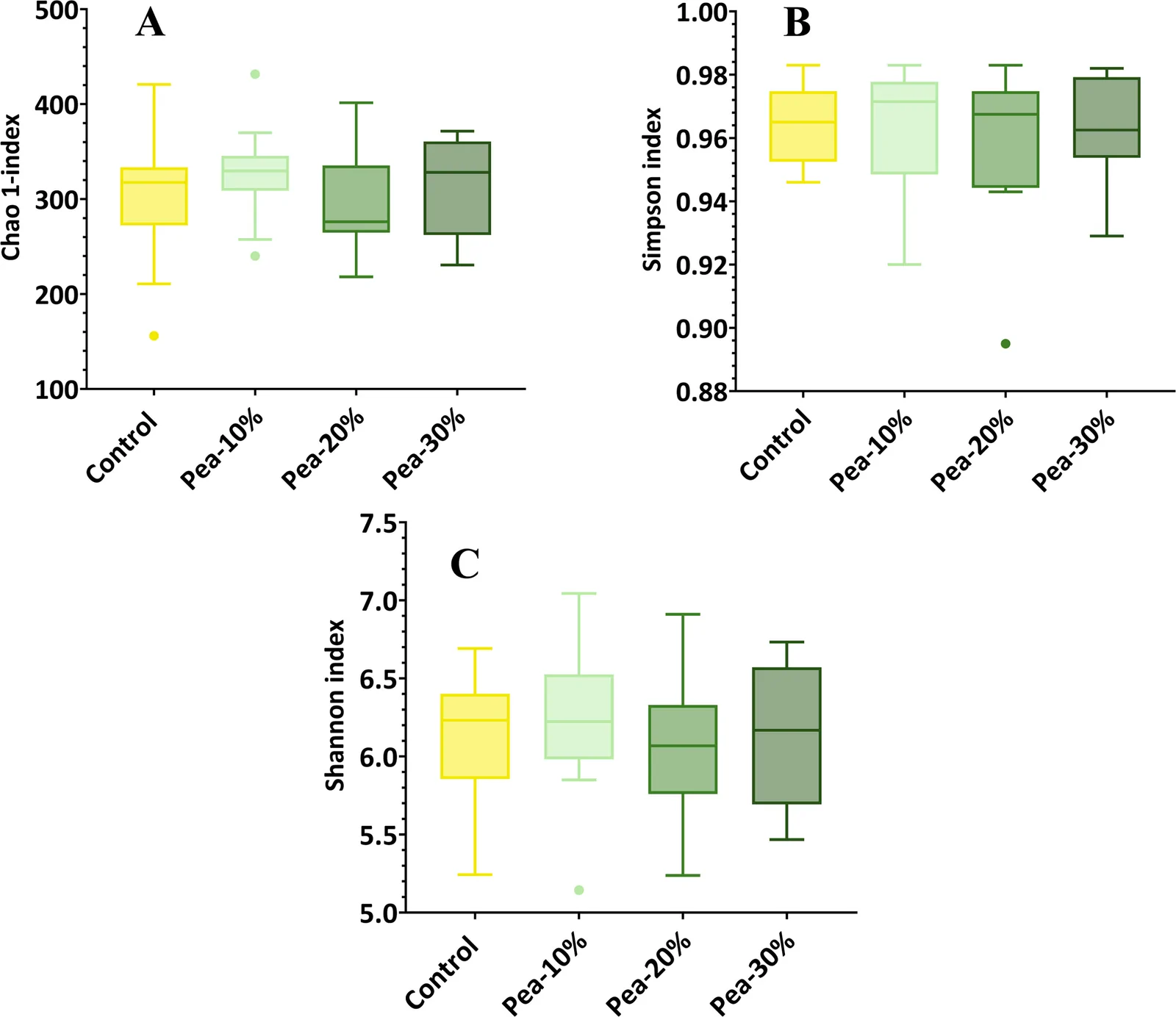
Alpha-diversity (A: Chao1-index, B: Simpson index, C: Shannon index) across the four experimental diets (i.e., control, Pea 10%, Pea 20%, Pea 30%).

***Beta diversity.*** Principal coordinates analysis on Bray-Curtis distances is shown in Fig. 5. PERMANOVA showed a significant effect of diet on β-diversity. However, when performing pairwise PERMANOVA, no significant differences were detected between individual diets following FDR correction, possibly due to the low sample size.

**Fig. 5:**
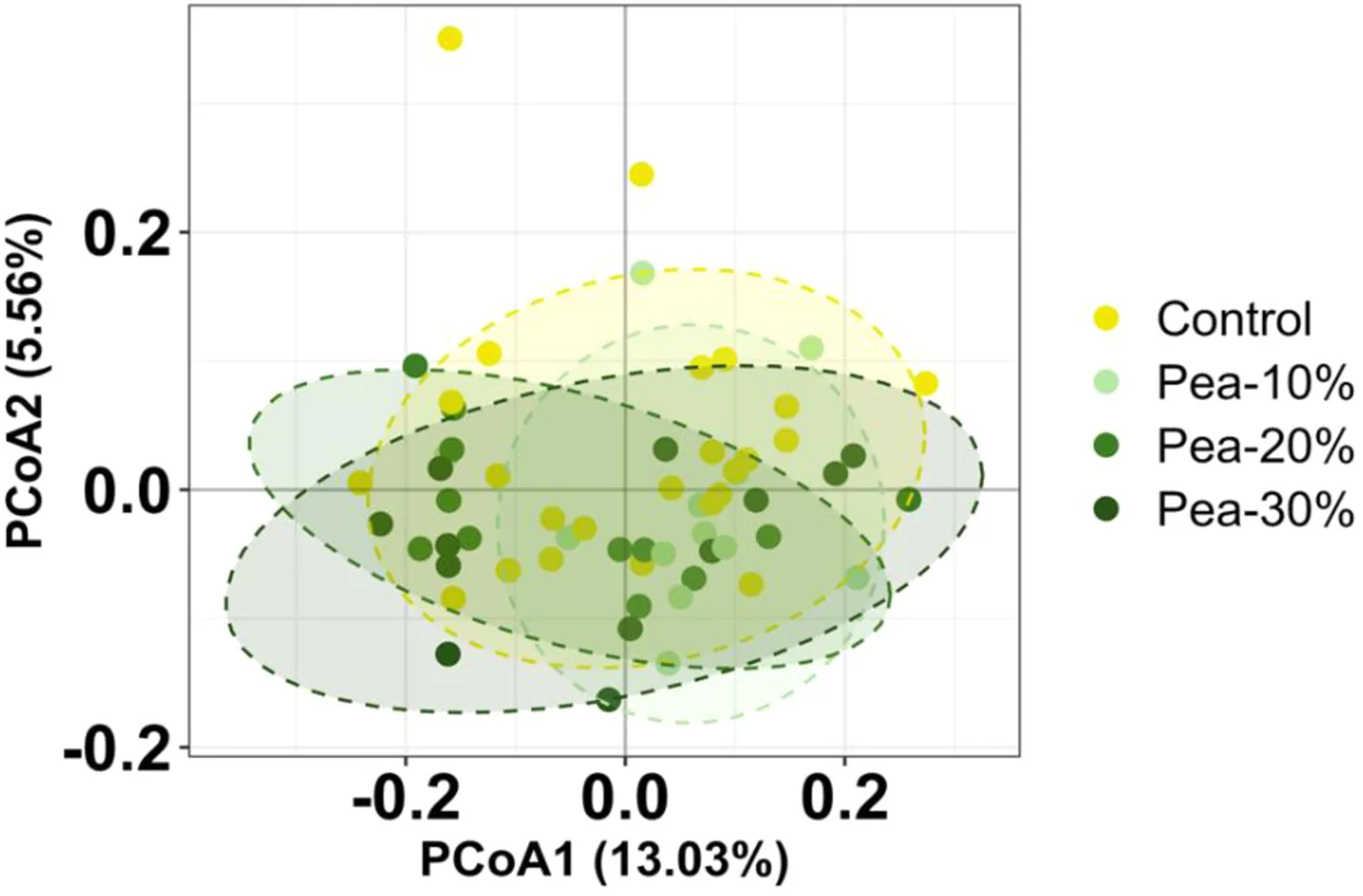
Principal coordinates analysis of beta diversity based on Bray-Curtis dissimilarity in caecal content across the four experimental diets (i.e., control, Pea 10%, Pea 20%, Pea 30%).

***Microbiota composition.*** In general, the same 10 genera were abundantly present in caecal contents of broilers fed the four different diets (Fig. 6A). Mainly *Lactobacillus* was present, followed by *Faecalibacterium*, and *Mediterraneibacter*. LEfSe analysis revealed a limited number of discriminant bacterial genera in the caecal microbiota of broilers (Fig. 6B). At the genus level, *Romboutsia* was identified as a biomarker for the Pea 30% treatment, with the highest LDA score among the detected taxa. In contrast, *Catellicoccus* was enriched in the control group. No discriminant genera were detected for the Pea 10% or Pea 20% treatments.

**Fig. 6:**
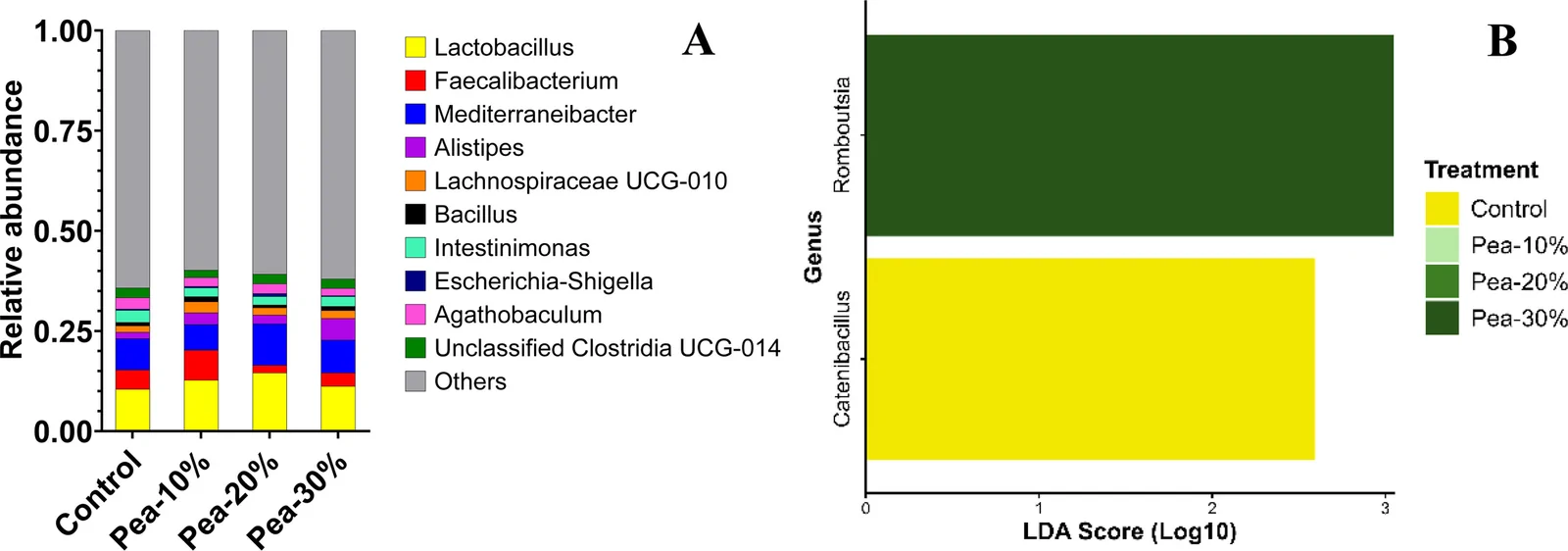
Relative microbial abundance of the 10 major genera (A) and Linear discriminant analysis Effect Size (LEfSe) analysis identifying differentially abundant genera (B) in caecal content across the four experimental diets (i.e., control, Pea 10%, Pea 20%, Pea 30%).

### In vitro simulation of the young broiler total gastrointestinal tract

#### Increasing pea inclusions influence in vitro protein digestion kinetics in the upper GIT

Fig. 7A shows the relative amount of readily bio-accessible protein as a function of small intestinal digestion time, representing the protein digestion kinetics modeled using a fractional conversion model. Estimated kinetic parameters *k* and *C_f_* are presented in Table 4. All experimental diets showed an initial increase in protein digestion, followed by a plateau. In vitro protein digestion kinetics of the control and Pea 10% diet were significantly different from those of the Pea 20% and Pea 30% diets, as indicated by the joint confidence regions in Fig. 7B. Pea 20% and Pea 30% diets especially exhibited lower *k* values and quickly reached lower levels of in vitro protein digestion during simulated small intestinal digestion. Overall, a downward trend in protein digestibility was observed in vitro from the control diet toward diets with higher pea inclusion rates, which is consistent with the same downward trend observed in vivo.

**Fig. 7:**
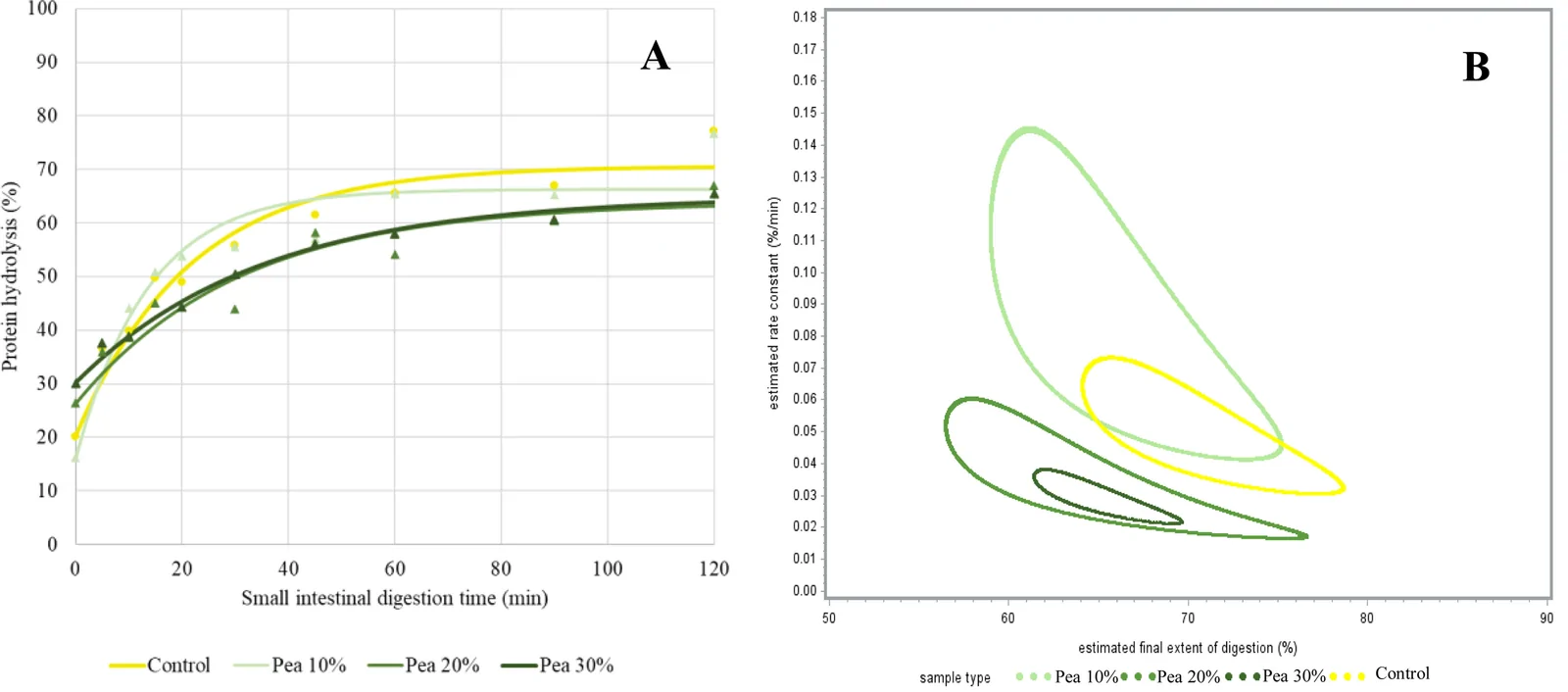
In vitro protein hydrolysis kinetics in the broiler small intestinal phase modeled using a fractional conversion model (Equation (3)) (A) and joint confidence region analysis of the kinetics (B) of the four experimental diets: Control (Yellow), Pea 10% (Light Green), Pea 20% (Green), and Pea 30% (Dark Green).

**Table 4:** Estimated kinetic parameters of in vitro protein digestion kinetics of the four experimental diets (Control, Pea 10%, Pea 20%, Pea 30%), with k (min^−1^) the rate constant and C_f_ the final extent, obtained using a fractional conversion model (equation (3)).

Diet	*k* (min^−1^)	*C_f_* (%)
**Control**	0.047 ± 0.007	70.58 ± 2.65
**Pea 10%**	0.074 ± 0.015	66.29 ± 2.89
**Pea 20%**	0.032 ± 0.007	63.90 ± 3.29
**Pea 30%**	0.028 ± 0.003	64.97 ± 1.53

#### Differences in composition of the undigested fraction

It is known that a fraction of the nutrients might not be digested in the upper GIT. The nutrients remaining in this fraction and the other indigestible compounds (e.g., fibers) are therefore available to the present microbiota during fermentation. Although research often does not analyze the composition of this fraction, it is essential to understand the differences in nutritional composition among the undigested fractions of the diets. The protein and NDF, ADF, and ADL content of the lyophilized undigested fractions are depicted in Table 5. It was apparent that the undigested fraction of the control diet contained relatively significantly more protein (11.31%), than the Pea 20% and Pea 30% diets (8.47-9.07%). Although control showed a higher relative protein content in the undigested residue, this did not indicate lower protein digestibility. The total dry mass of the residue was lower in comparison to the pea-containing diets, suggesting more extensive digestion and/or solubilization of the overall matrix. Consequently, the remaining residue may be relatively enriched in protein due to the preferential removal of non-protein components such as starch or soluble carbohydrates. Moreover, NDF and ADL fractions significantly decreased with increasing pea fractions, while ADF values significantly increased. When hemicellulose and cellulose were estimated from the detergent fiber fractions, hemicellulose showed an overall decrease, whereas calculated cellulose increased numerically. This suggests that replacing soybean meal with pea shifted the residual cell wall fraction toward a more cellulose-dominated and less lignified (cfr., ADL) composition.

**Table 5:** Protein, NDF, ADF, and ADL content (DM basis) in the in vitro undigested fractions of the four experimental diets (i.e., control, Pea 10%, Pea 20%, and Pea 30%). Different letters in superscript indicate significant differences between the diets (P < 0.05).

	Protein (%)	NDF (%)	ADF (%)	ADL (%)
**Control**	11.31 ± 0.47^a^	74.51 ± 3.94^a^	29.89 ± 1.68^a^	3.46 ± 1.02^a^
**Pea 10%**	10.37 ± 0.08^a^	65.52 ± 3.84^b^	30.11 ± 4.41^a^	2.29 ± 0.63^ab^
**Pea 20%**	9.07 ± 0.35^b^	69.93 ± 2.64^ab^	33.79 ± 1.25^a^	0.89 ± 0.47^b^
**Pea 30%**	8.47 ± 0.13^b^	65.08 ± 0.96^b^	34.67 ± 0.49^a^	0.55 ± 0.52^b^

#### In vitro gas production kinetics increased by pea inclusion in the lower GIT

Subsequently, the undigested fraction reaches the lower GIT, where it can be fermented. For this purpose, the undigested fraction obtained after simulating the upper GIT was fermented using broiler caecal content. The in vitro gas production profiles are depicted in Fig. 8, estimated model parameters are shown in Table 6. First of all, fermentation of the control diet resulted in a significantly lower total gas production (*A*) and half asymptote time (*B*) in comparison to all diets containing pea. Similarly, the control diet resulted in a significantly lower maximum gas production rate (*R_max_*), and this rate was reached significantly earlier (*T_max_*) in comparison to all pea-containing diets, with Pea 30% even reaching a significantly higher *R_max_* than the remaining two pea diets.

**Fig. 8:**
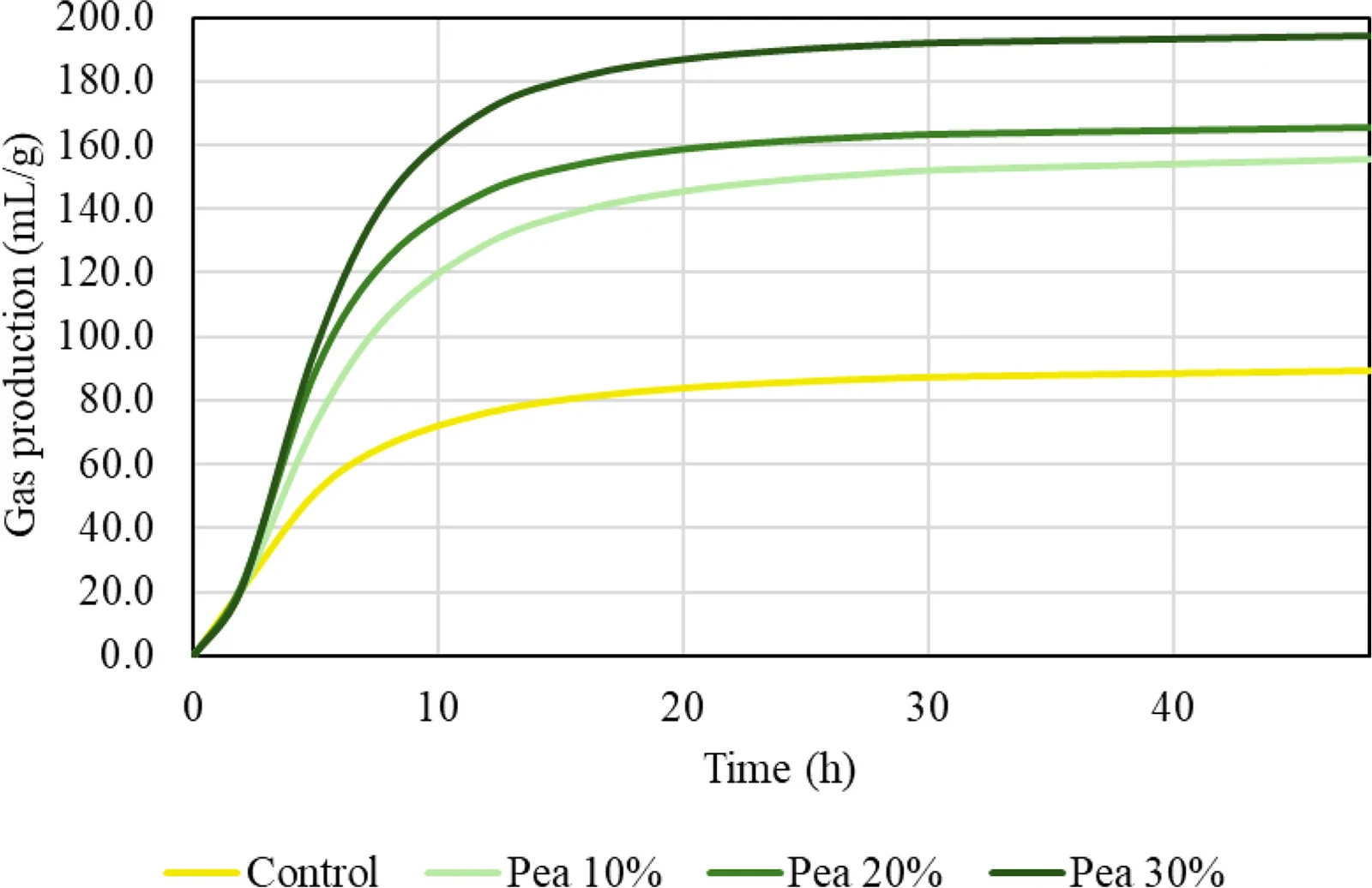
In vitro fitted cumulative gas production kinetics in the broiler lower GIT using a Groot monophasic model (mL/g) of the four experimental diets: Control (Yellow), Pea 10% (Light Green), Pea 20% (Green), and Pea 30% (Dark Green).

**Table 6:** Estimated kinetic parameters of the in vitro fitted cumulative gas production kinetics in the broiler lower GIT using a Groot monophasic model (Equation (4), mL/g) of the four experimental diets.

	Control	Pea 10%	Pea 20%	Pea 30%
***A***	91.17 ± 28.86^b^	158.29 ± 26.34^a^	167.94 ± 13.23^a^	195.35 ± 21.44^a^
***B***	4.21 ± 0.67^b^	5.42 ± 0.26^a^	5.30 ± 0.11^a^	5.05 ± 0.41^a^
***R_max_***	13.07 ± 2.88^c^	18.63 ± 1.77^a^	21.23 ± 0.78^a^	27.04 ± 1.03^b^
***T_max_***	1.69 ± 0.22^b^	2.91 ± 0.44^a^	3.27 ± 0.18^a^	3.32 ± 0.41^a^

#### In vitro SCFA and BCFA production driven by pea fractions

Fig. 9 shows the SCFA production during in vitro fermentation at 6, 12, 24, and 48 h. Evidently, SCFA production increased over the course of fermentation. In general, acetic acid was produced in the highest amounts across all diets, followed by propionic acid and butyric acid. Additionally, significantly less acetic, propionic, and butyric acid was formed after 48 h when fermenting the undigested fraction of the control diet, in contrast to the pea 30% diet, with concentrations of 24.6, 5.9, and 6.7 mg/mL substrate of the control diet, respectively. Contrarily, pea-containing diets formed concentrations ranging between 34.8-40.2, 8.2-9.1, and 7.3-10.8 mg/mL substrate of acetic, propionic, and butyric acid, respectively. Thus, SCFA production showed a clear increasing trend with increasing incorporation of the pea fraction. Consistent with these findings, a significant downward trend was observed for BCFA production, including iso-butyric acid and iso-valeric acid.

**Fig. 9:**
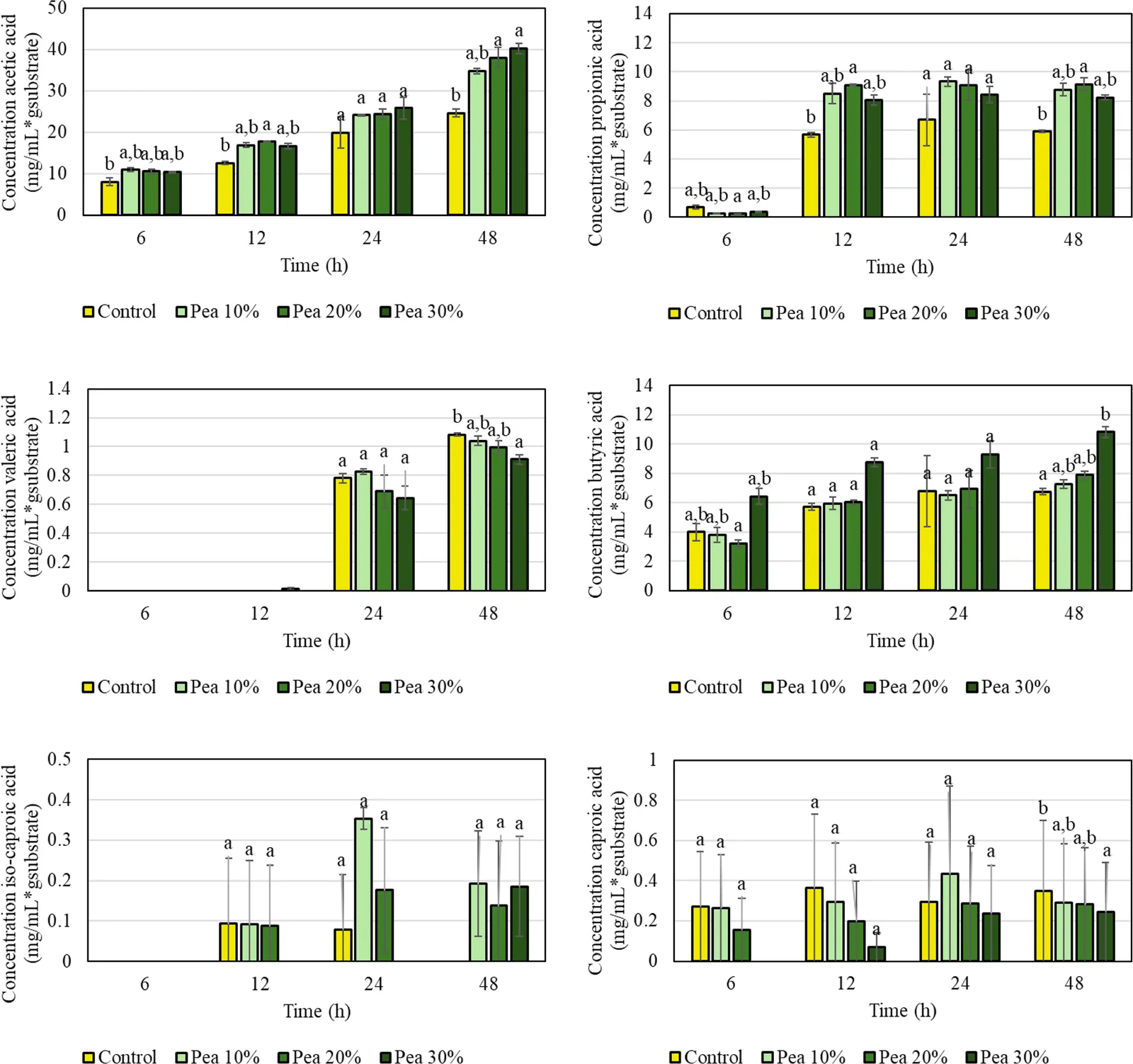
In vitro short-chain fatty acid production in the broiler simulated lower GIT (mg/mL*g substrate) of the four experimental diets at 6, 12, 24, and 48 h of in vitro fermentation: Control (Yellow), Pea 10% (Light Green), Pea 20% (Green), and Pea 30% (Dark Green).

#### Lower in vitro ammonia production in pea containing feeds

Ammonia production at 6, 12, 24, and 48 h during fermentation is depicted in Fig. 10. Similar to SCFA production, ammonia production increased with in vitro fermentation time. While differences among diets were small after 6 h, they became more pronounced as fermentation progressed. Although the differences were not statistically significant, ammonia production increased more strongly in the control diet and showed a progressively slower increase as pea inclusion increased. Consequently, a clear downward trend was observed from the control diet to the Pea 30% diet, with final ammonia production values of 6.1 and 4.4 mL mL⁻¹ g⁻¹ substrate, respectively.

**Fig. 10:**
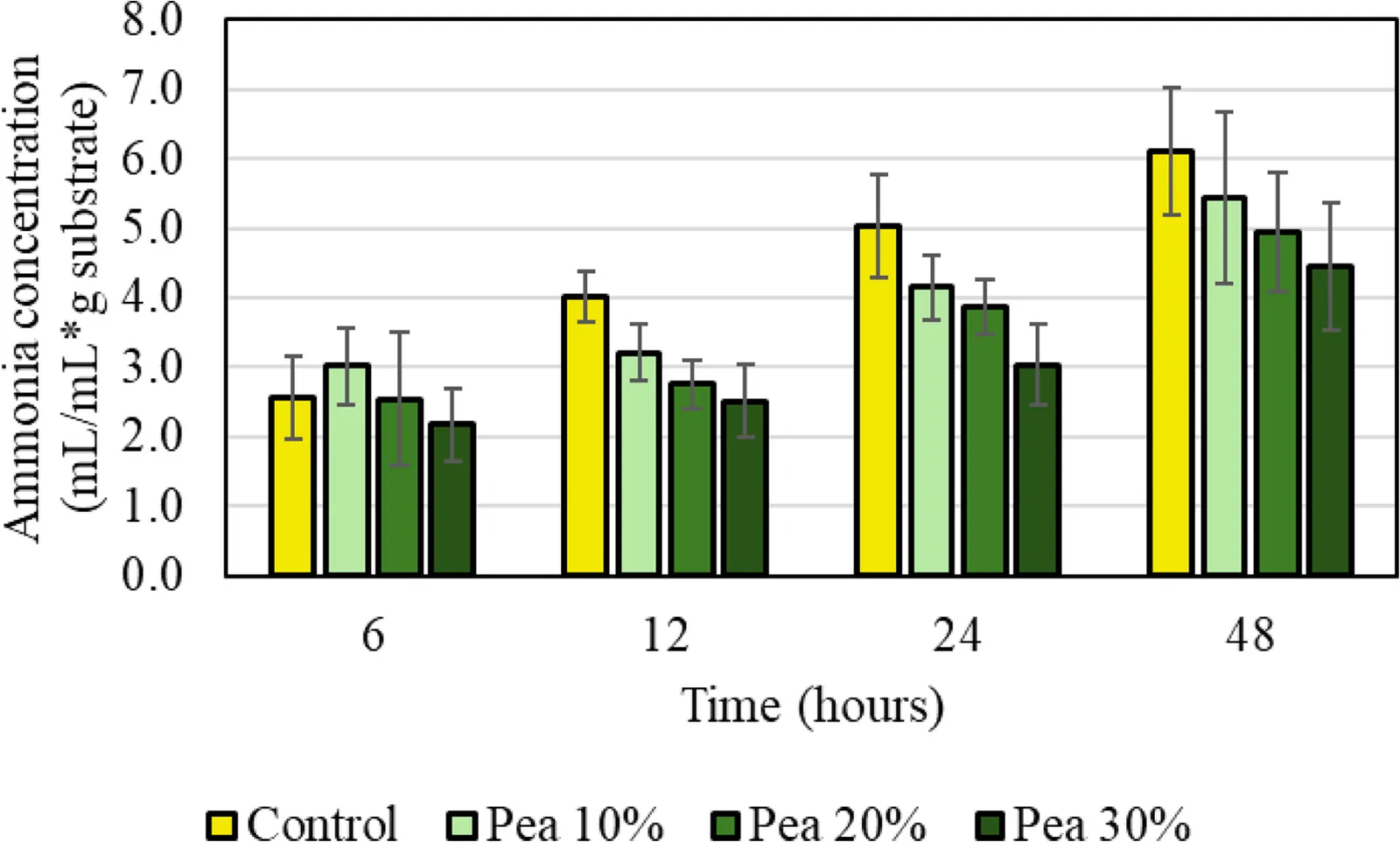
In vitro ammonia production in the broiler simulated lower GIT (mg/mL*g substrate) of the four experimental diets at 6, 12, 24, and 48 h of in vitro fermentation: Control (Yellow), Pea 10% (Light Green), Pea 20% (Green), and Pea 30% (Dark Green). (P > 0.05).

#### In vitro microbial composition affected by highest pea inclusion

***Alpha-diversity.*** Fig. 11 shows the Chao1-, Simpson-, and Shannon indices as a measure for α-diversity. Similar to the in vivo results, the α-diversity was not significantly different across diets. However, while not significant, a clear downward trend was again observed with increasing incorporation of peas in the diets, especially in the Simpson and Shannon indices describing how evenly different genera are distributed within the microbial population. Additionally, one clear outlier was present in the control group.

**Fig. 11:**
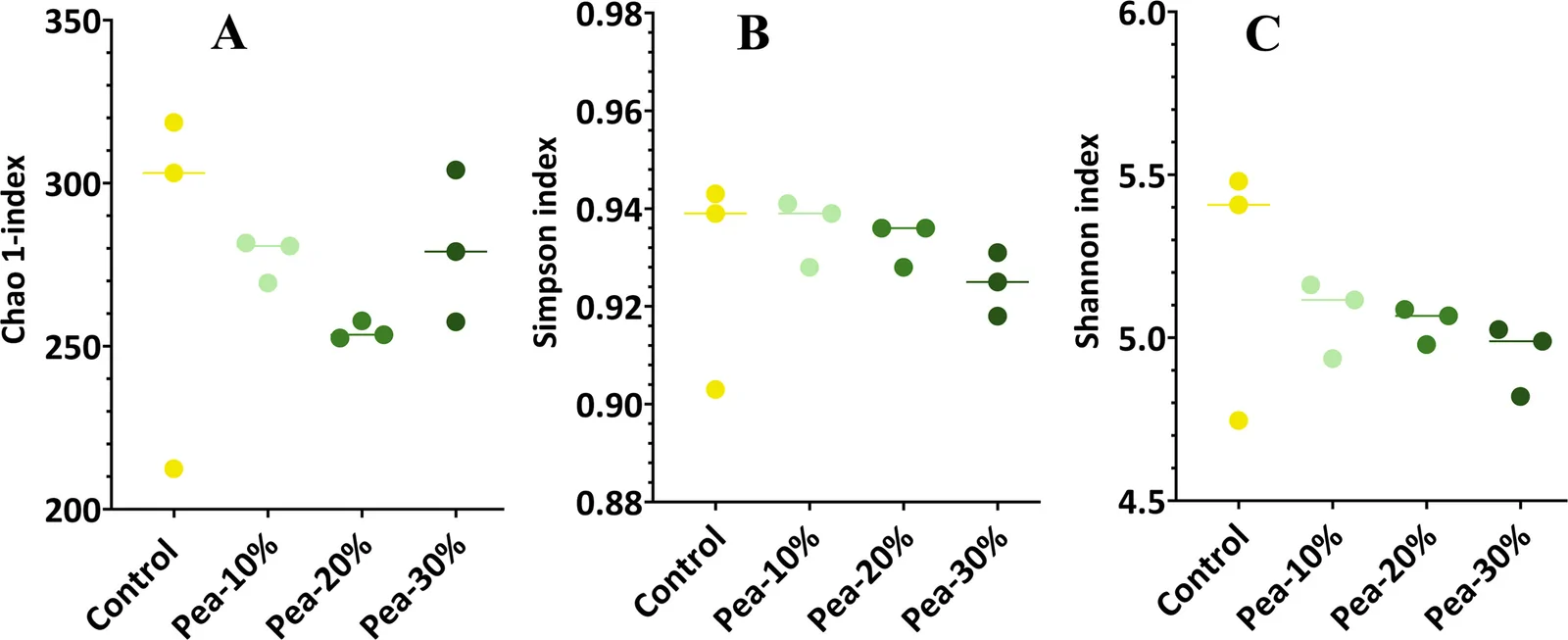
Alpha-diversity (A: Chao1-index, B: Simpson index, C: Shannon index) across the four experimental diets (i.e., control, Pea 10%, Pea 20%, Pea 30%) after 12 h of in vitro fermentation mimicking the young broiler lower GIT.

***Beta-diversity.*** Fig. 12 shows the β-diversity of the inoculum and four experimental diets. PERMANOVA revealed a significant effect of diet on β-diversity. Pairwise PERMANOVA further showed a significant difference in β-diversity between the control diet and the Pea 30% diet, thereby revealing that the microbial composition in the caeca of broilers was significantly different in young broilers fed the respective diets. Similar to the previously described in vivo results, PERMANOVA indicated a significant effect of diet on β-diversity.

**Fig. 12:**
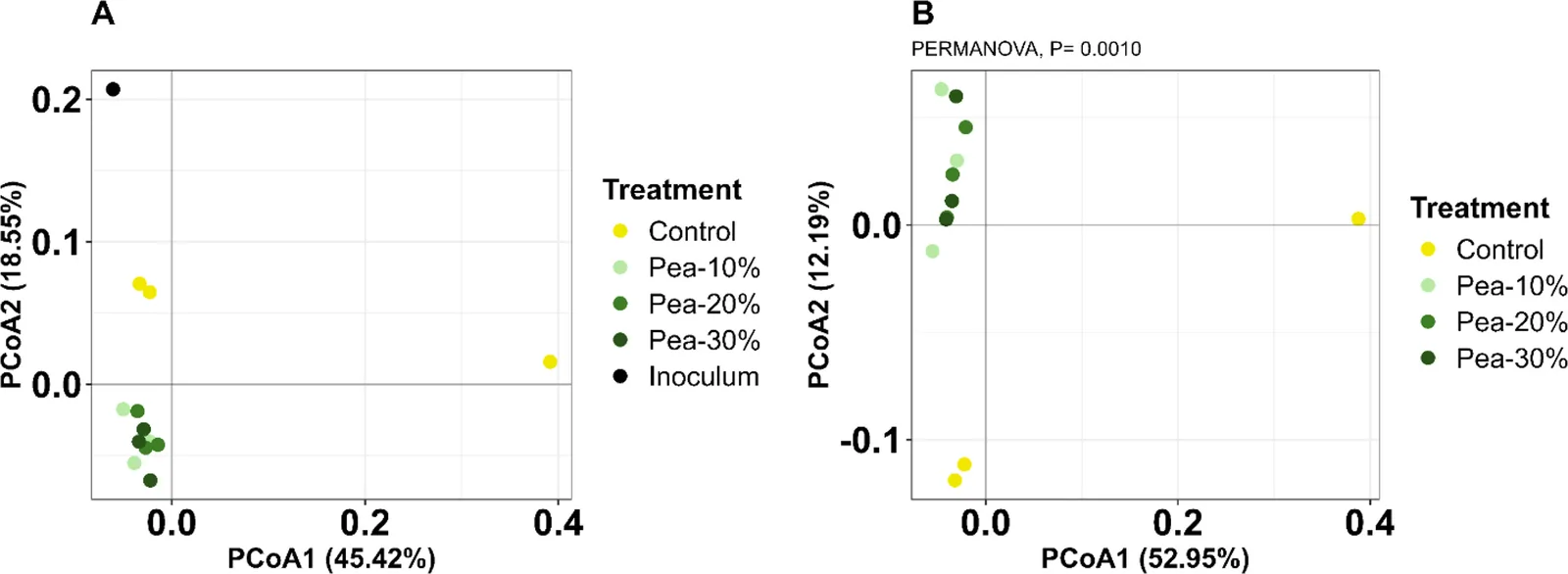
Principal coordinates analysis of beta-diversity based on Bray-Curtis dissimilarity after 12 h of in vitro fermentation of the four experimental diets (i.e., control, Pea 10%, Pea 20%, Pea 30%) (A) with inoculum, and (B) without inoculum.

***Microbiota composition.*** The top 10 genera present in the in vitro fermentation samples after 12 h are shown in Fig. 13A. Mainly Bifidobacterium was present, followed by *Streptococcus*, and *Blautia*. In line with larger differences in β-diversity and previous in vivo results, LEfSe analysis revealed a number of discriminant bacterial genera in the in vitro fermentation samples in the control, one for the Pea 20% diet and one for the Pea 30% diet (Fig. 13B). At genus level, *Faecalibacterium* was identified as a biomarker for the Pea 30% treatment. In contrast, *Ruminococcaceae* was enriched in the control group. Streptococcus was a discriminant genus for the Pea 20% diet. No discriminant genera were detected for the Pea 10% diet.

**Fig. 13:**
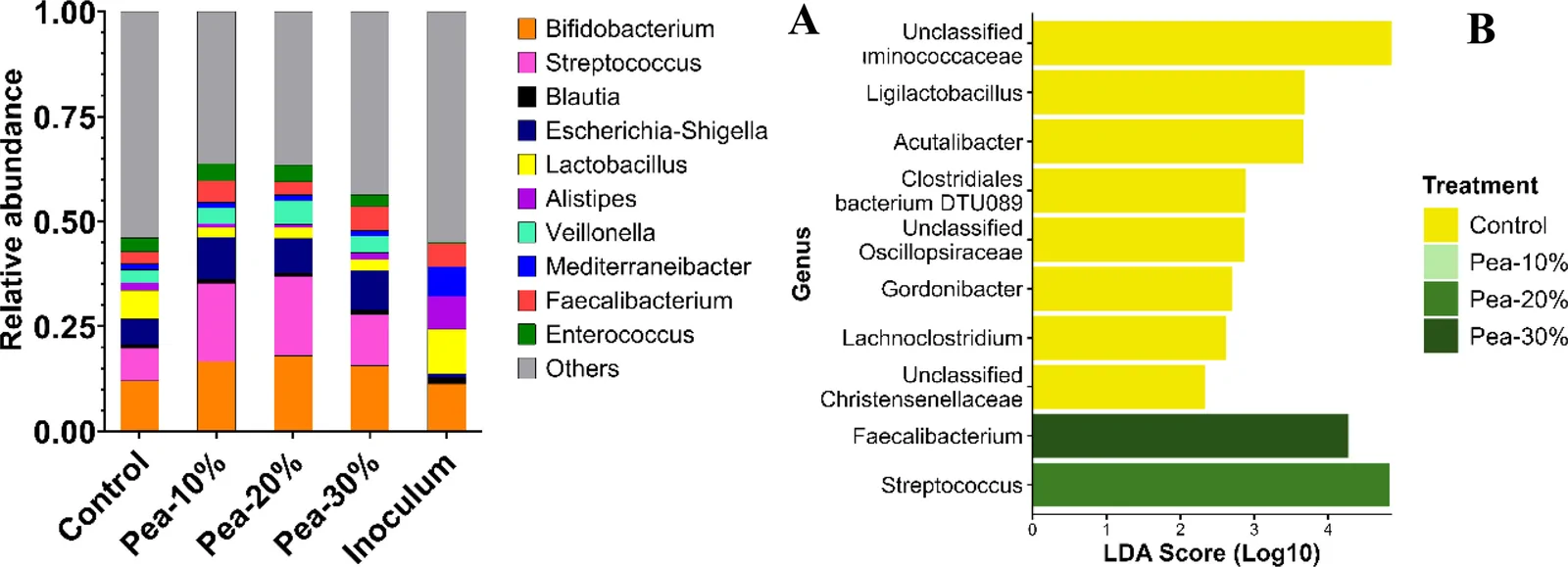
Relative microbial abundance of the 10 major genera (A) and Linear discriminant analysis Effect Size (LEfSe) analysis identifying differentially abundant genera (B) in in vitro fermentation samples after 12 h across the four experimental diets (i.e., control, Pea 10%, Pea 20%, Pea 30%).

## Discussion

In this study, a standardized, integrated in vitro model was proposed to simulate the complete gastrointestinal tract of young broilers, including gastric digestion, small intestinal digestion, absorption and caecal fermentation under conditions selected to reflect key physiological characteristics of starter broilers. Using this model in parallel with an in vivo trial, raw peas were evaluated as a partial alternative protein source to SBM in diets for young broilers. The parallel use of both approaches enabled comparison of the in vitro responses with the in vivo findings, thereby providing the data to assess the extent to which comparable digestive and fermentative response patterns were observed between both approaches and to evaluate the physiological relevance of the in vitro model. At the same time, the two approaches should be considered complementary rather than interchangeable. Whereas the in vivo trial reflects the integrated response of the animal, including growth performance, nutrient utilization, intestinal morphology, and caecal microbial ecology, the in vitro model enables a more controlled, time-dependent assessment of digestion and fermentation responses. Combining both approaches, therefore, provides a more comprehensive, complementary evaluation of raw pea inclusion.

### In vivo broiler trial

The differences observed in AID between the SBM-feed and feeds containing pea could be attributed to combined ingredient-matrix and processing effects. SBM is a dehulled, oil-extracted, heat-treated protein source, whereas raw whole peas provide protein embedded in a starch–fiber matrix and may contain residual antinutritional factors (Herkelman et al., 1993; Wu et al., 2023). Therefore, lower protein digestion in pea-containing diets is likely caused by a combination of protein structure, very mild processing, fiber effects, and antinutritional factors, as the diet used was a mash diet. The reduced broiler performance and AID observed with increasing pea inclusion levels were in accordance with previous literature. Sadr et al. (2026) reported no significant differences in 10-day-old broilers fed a diet containing 12% peas compared with an SBM-based diet. Contrarily, Biesek et al. (2020) observed a significant reduction in final body weight in 6-week-old broilers fed a diet in which SBM was partially replaced by 25% peas. Similarly, Moschini et al. (2005) reported a decrease in average daily gain in 21-day-old broilers fed diets containing 17% or 35% peas compared with an SBM control diet. Although several studies have investigated the effects of pea inclusion on broiler growth performance, knowledge on its effects in starter broilers remains limited.

While the effects of pea inclusion in broiler diets have received increasing attention over the past decades, only limited research has reported ileal digestibility values for pea-containing diets, particularly in young broilers. Indeed, available studies have often focused on older birds, and digestibility values of pea-containing diets have not always been compared with those of a control diet. Hamungalu et al. (2026) reported a significant decrease in standardized ileal CP digestibility, from 90.7% to 82.2%, when SBM was completely replaced by peas in diets for 24-day-old broilers. Overall, the apparent ileal CP digestibility values observed in the present study, as well as the decreasing trend with increasing pea inclusion levels, are consistent with previous reports in the literature.

A significant decrease in VH was in line with the study by Röhe et al. (2017) in 35-day-old broilers fed a diet containing raw peas in comparison to a SBM control diet, although measured in jejunal samples rather than ileal samples. Jha & Mishra (2021) suggested that raw peas can indeed cause such abrasion of the mucosal surface, as an excess of dietary fibers can significantly abrade the mucosal surface, thereby shortening the villi. Moreover, a substantial decrease in VH could decrease the potential of broilers to absorb nutrients (Jha and Mishra, 2021). Therefore, the reduced VH in ileal samples of broilers fed the Pea 30% could be a secondary reason why less protein was digested in comparison to the control.

Caecal fermentation of the different feeds, assessed by SCFA and ammonia concentrations, was not significantly affected in 10-day-old broilers. This limited metabolite response is in line with previous broiler research. Indeed, Kirn et al. (2024) reported that diets containing peas, pea protein concentrate, or pea hulls did not significantly affect caecal SCFA concentrations, while effects on the caecal microbiota were restricted mainly to subdominant genera. Similarly, in the present study, LEfSe analysis revealed only a limited number of discriminant bacterial genera, which may also be partially attributed to the broilers’ young age. *Romboutsia* was enriched in the Pea 30% treatment, and *Catellicoccus* was enriched in the control group, whereas no discriminant genera were detected for the Pea 10% or Pea 20% treatments. This may indicate that only the highest pea inclusion level provided sufficient substrate to induce a detectable taxonomic shift. The enrichment of *Romboutsia* in the Pea 30% treatment may reflect a substrate-driven response to pea-derived carbohydrate fractions, which are hypothesized to include starch, resistant starch, or oligosaccharides, entering the caeca. *Romboutsia* is part of the normal intestinal microbiota of broilers and has been associated with carbohydrate utilization (Gerritsen et al., 2017). When fermentable carbohydrates are available, microbial fermentation typically shifts preferentially toward saccharolytic pathways, with reduced reliance on protein fermentation (Elling-Staats et al., 2022). Therefore, the enrichment of *Romboutsia* in the Pea 30% treatment may indicate an adaptation of the caecal microbiota to the greater availability of fermentable pea-derived substrates compared with the SBM-based control diet.

Overall, the in vivo results indicate that increasing pea inclusion mainly affected protein digestion and selected aspects of intestinal morphology, while caecal fermentation end-products remained largely unchanged. The limited caecal metabolite response, together with the restricted number of discriminant bacterial genera, suggests that pea inclusion induced only subtle microbial adaptations in 10-day-old broilers.

### In vitro broiler GIT simulation

While in vivo protein digestibility values provided a direct insight into the effect of pea addition to feed on endpoint digestion and fermentation, they gave little insight into which factors might have influenced differences in digestion and fermentation. For this purpose, performing in vitro GIT simulations can offer complementary insights by evaluating protein digestion kinetics and fermentation.

Differences in in vitro digestion kinetics between the control diet and the diet containing 10% of peas on the one hand, and diets containing 20% and 30% peas on the other hand, could be attributed to a major difference in the processing histories of SBM and peas. While soybeans were dehulled and heat-treated to obtain SBM, peas were raw-milled. Research showed that dehulling and thermal processing of pulses reduced the quantity of important antinutrients such as polyphenols and trypsin inhibitors, respectively. Subsequently, dehulling and thermal processing significantly improved protein digestion (Staes et al., 2026). Staes et al. (2026) demonstrated that this can significantly impact the rate of protein digestion, while still obtaining similar final extents. Therefore, the significantly different protein digestion kinetics in the present study provided additional insights into how peas might have resulted in a lower protein digestibility observed in the in vivo trial. Additionally, these in vitro results, simulating the young broiler upper GIT, showed the same trends observed in the in vivo experiment.

The higher gas production observed for the Pea 30% treatment during in vitro fermentation is likely related to the greater availability of fermentable fibers after gastric and small-intestinal digestion. Research has reported that when fibers become scarcer, protein is preferentially fermented, resulting in lower gas production (Elling-Staats et al., 2022). While peas and SBM were reported in literature to contain approximately equal amounts of fermentable fibers (Knudsen, 2014), it is important to note that in the present study, a fraction of wheat has been replaced by peas as well (Table 1). Whole peas contain C-type starch, which, in its native state, is less digestible than A-type wheat starch (Zhang et al., 2006). Accordingly, it was hypothesized that more undigested resistant starch was present in digested pea-containing diets compared with the control, resulting in higher rates and extents of gas production. Higher caecal fiber fermentation may contribute to improved gut health in broilers by promoting short-chain fatty acid production and supporting a more favorable intestinal microbial environment. Therefore, these parameters were also analyzed.

Additionally, the significantly higher SCFA and numerically, but non-significantly lower ammonia formation in the Pea 30% diet in comparison to the control also suggested that increasing pea inclusion shifted toward a more saccharolytic fermentation. The LEfSe results support this interpretation by showing enrichment of *Faecalibacterium* in the Pea 30% treatment. *Faecalibacterium* is a common member of the broiler caecal microbiota and includes butyrate-producing bacteria, which is consistent with the higher butyrate formation observed in the Pea 30% fermentation. Moreover, the more abundant presence of *Faecalibacterium* was also observed by other researchers as a result of resistant starch fermentation (Haenen et al., 2013; Regassa and Nyachoti, 2018). Similarly, in the present study, it was hypothesized that larger fractions of resistant starch were present in the undigested fraction of diets containing increasing amounts of peas, as discussed above.

### Capability of the newly defined in vitro model simulating the full GIT of young broilers

Overall, comparison of the in vivo and in vitro results showed that the proposed innovative in vitro model reflected several key digestive response patterns observed in young broilers, while fermentative responses were more pronounced under in vitro conditions. Although the caecal fermentation responses were more pronounced in vitro than in vivo, the overall direction of the observed changes was comparable, suggesting that the controlled in vitro system may reveal dietary effects that are less readily detected in vivo due to biological variability and host-related factors. For example, an advantage of the in vitro fermentation model is its ability to directly assess microbial fermentation capacity through SCFA production, without the confounding effects of intestinal SCFA absorption that occur in vivo. Additionally, in vitro systems operate under highly standardized and controlled conditions, reducing experimental variability and increasing sensitivity for detecting shifts in, e.g., digestion kinetics and fermentation patterns. As a result, in vitro models may reveal dietary responses that are more difficult to establish statistically in vivo, even when the overall direction of the response is comparable. As such, the model represents a valuable tool for the screening of feed ingredients or formulations prior to labor-intensive and ethically challenging in vivo evaluation, allowing feed ingredients or additives with promising nutritional responses to be identified at an early stage and prioritized for subsequent in vivo evaluation. In addition, the model can complement in vivo trials by enabling more detailed investigation of digestion- and fermentation-related processes over time (i.e., kinetics). This provides mechanistic insight that is difficult to obtain from endpoint measurements in vivo, thereby supporting the use of the model as both a screening and complementary explanatory tool in young-broiler nutrition research.

## Conclusion

This study shows that raw peas can contribute to more sustainable broiler diets, but only within limits for young broilers. Higher raw pea inclusion reduced growth performance, protein digestibility, and ileal villus height, indicating impaired nutrient utilization during a life stage in which digestive capacity is still developing. This underscores the importance of age-adapted in vitro models, as young broilers have lower digestive maturity and enzyme secretion, and their caecal microbiota is still being established. The newly developed in vitro model showed agreement with key in vivo responses, particularly for protein digestion, while providing complementary, time-dependent insight into digestion and fermentation kinetics. However, agreement was more limited for caecal fermentation, where increased gas and SCFA production, reduced ammonia formation, and changes in microbial composition observed in vitro were more pronounced than in vivo. Nevertheless, these findings support the relevance of a model that simulates the complete gastrointestinal tract of young broilers, from gastric digestion to small intestinal digestion and caecal fermentation, which, to our knowledge, has not previously been integrated within a single in vitro model for young broilers. Thus, the model represents a promising tool for rapid, physiologically informed, and controlled screening of feed ingredients and formulations for early-life broiler nutrition prior to animal testing. However, to further improve the model, future research should investigate how caecal inoculum selection and preparation affect fermentation outcomes, and the effect of using only soluble components and fine particles of the undigested fraction for caecal fermentation, as these two factors were hypothesized to overexpress in vitro outcomes. Moreover, future work could also include analysis of amino acid profiles in ileal samples and in vitro small intestinal digests, providing more detailed insight into protein quality and amino acid availability. Finally, this model provides a controlled, high-throughput approach that may be applied to the evaluation of feed ingredients and feed additives, including future alternative protein sources such as protein-rich plant-based waste streams or side streams. Such ingredients are often already heat-treated, which may reduce antinutritional factors compared with raw peas, thereby potentially improving their suitability as sustainable protein sources. Overall, this study demonstrates the potential of the developed full-GIT young broiler in vitro model as a complementary screening tool, particularly for assessing protein digestion responses. Although the caecal fermentation stage provides valuable insight into microbial fermentation potential, further optimization and validation against in vivo fermentation endpoints are required to establish its predictive capacity.

## Author Contributions

**Daphne Michels**: Conceived and designed the study, developed the methodology, conducted experiments, analyzed and interpreted the data, and drafted the manuscript.

**Nona de Groof**: Conducted experiments, collected and analyzed data, and contributed to their interpretation and critical review of the manuscript.

**Sarah H.E. Verkempinck**: Contributed to the study conception and methodological design and critically reviewed the manuscript.

**Evelyne Delezie**: Contributed to the acquisition of experimental data and critically reviewed the manuscript.

**Muhammad Zeeshan Akram**: Performed and interpreted data analyses and critically reviewed the manuscript.

**Nadia Everaert**: Conceived and supervised the study, contributed to the interpretation of results, secured funding, and critically revised the manuscript.

**Tara Grauwet**: Conceived and supervised the study, contributed to the interpretation of results, secured funding, and critically revised the manuscript.

All authors critically reviewed and explicitly approved the final version of the manuscript and agree to be personally accountable for all aspects of the work, ensuring that any questions concerning its accuracy or integrity are appropriately investigated and resolved.

## Funding

The project was funded by a KU Leuven C3-project (C3/23/020), and Dr Muhammad Zeeshan Akram is a postdoctoral researcher funded by a KU Leuven Postdoctoral Mandate (PDMT2/25/048).

## Disclosures

The authors of the present work declare no conflict of interests.
